# An Efficient Evaluation System Accelerates α-Helical Antimicrobial Peptide Discovery and Its Application to Global Human Genome Mining

**DOI:** 10.3389/fmicb.2022.870361

**Published:** 2022-04-25

**Authors:** Licheng Liu, Caiyun Wang, Mengyue Zhang, Zixuan Zhang, Yingying Wu, Yixuan Zhang

**Affiliations:** School of Life Sciences and Biopharmaceutics, Shenyang Pharmaceutical University, Shenyang, China

**Keywords:** antimicrobial peptide, screening system, antimicrobial activity, antimicrobial mechanism, mouse model, skin infection, physicochemical parameter, human genome

## Abstract

Antimicrobial peptides (AMPs), as an important part of the innate immune system of an organism, is a kind of promising drug candidate for novel antibiotics due to their unique antibacterial mechanism. However, the discovery of novel AMPs is facing a great challenge due to the complexity of systematic experiments and the poor predictability of antimicrobial activity. Here, a novel and comprehensive screening system, the Multiple Descriptor Multiple Strategy (MultiDS), was proposed based on 59 physicochemical and structural parameters, three strategies, and four algorithms for the mining of α-helical AMPs. This approach was applied to mine the encrypted peptide antibiotics from the global human genome, including introns and exons. A library of approximately 70 billion peptides with 15–25 amino acid residues was screened by the MultiDS system and generated a list of peptides with the Multiple Descriptor Index (MD index) scores, which was the core part of the MultiDS system. Sixty peptides with top MD scores were chemically synthesized and experimentally tested their antimicrobial activity against 10 kinds of Gram-positive bacteria, Gram-negative bacteria (including drug-resistant pathogens). A total of fifty-nine out of 60 (98.3%) peptides exhibited antimicrobial activity (MIC ≤ 64 μg/mL), and 24 out of 60 (40%) peptides showed high activity (MIC ≤ 2 μg/mL), validating the MultiDS system was an effective and predictive screening tool with high hit rate and superior antimicrobial activity. For further investigation, AMPs S1, S2, and S3 with the highest MD scores were used to treat the skin infection mouse models *in vivo* caused by *Escherichia coli*, drug-resistance *Escherichia coli*, and *Staphylococcus aureus*, respectively. All of S1, S2, and S3 showed comparable therapeutic effects on promoting infection healing to or even better than the positive drug levofloxacin. A mechanism study discovered that rapid bactericidal action was caused by cell membrane disruption and content leakage. The MultiDS system not only provides a high-throughput approach that allows for the mining of candidate AMPs from the global genome sequence but also opens up a new route to accelerate the discovery of peptide antibiotics.

## Introduction

With the increase of drug-resistant pathogens and the decline in the discovery of new antibiotics, the urgent priority is to develop new available antimicrobial drugs to fight against bacterial infectious diseases. Antimicrobial peptides (AMPs), as a promising alternative, have attracted extensive attention due to their wide antimicrobial spectrum and difficulty in developing drug resistance. As an important kind of AMP, α-helical AMPs are usually cationic linear peptides with amphipathic helical structures in contact with biological membranes (Wang et al., 2019). The antibacterial effect of AMPs is mainly through the destruction of microbial lipid bilayers to induce the leakage of cell contents (Avci et al., 2018; Kumar et al., 2018). The physicochemical properties and structure features, such as net charge (Jiang et al., 2008), charge density (Vishnepolsky and Pirtskhalava, 2014), hydrophobicity (Edwards et al., 2016; Lee et al., 2018), hydrophobic moment (Wieprecht et al., 1997b), amphiphilicity (Pathak et al., 1995), angle (Wieprecht et al., 1997a), length (Gagnon et al., 2017), helicity (Dathe et al., 1996; Huang et al., 2014), propensities to the disordered structure, and aggregation (Fernandez-Escamilla et al., 2004; Conchillo-Solé et al., 2007; De Groot and Ventura, 2010), are essential to exert antimicrobial activities. In recent years, computational approaches based on these parameters have been widely used in the discovery of AMPs, overcoming the difficulties in systematic experimental identification of AMPs due to the limitations of current methods. Torres et al. (2021) mined the encrypted peptide antibiotics in the human proteome *via* an algorithm that relied on the sequence length, net charge, and average hydrophobicity, and the results showed 63.6% of the encrypted peptides displayed antimicrobial activity against pathogens. Besides, many algorithms and models, such as quantitative structure-activity relationship (QSAR; Taboureau et al., 2006), support vector machines (SVM; Porto et al., 2012), random forests (RF; Joseph et al., 2012), discriminant analysis (DA), and artificial neural network (ANN; Fjell et al., 2009), have also been built and applied to predict and evaluate potential AMPs. Hou et al. (2019) applied a combination of three databases (AntiBP2, APD3, and CAMP_R3_) and four algorithms (SVM, RF, DA, and ANN) to predict AMPs from the protein hydrolyzate of Sichuan pepper seeds, one of the 16 potential AMPs with high scores exhibited moderate antibacterial activity against *Escherichia coli* ATCC 25922 with an MIC value of 64 μg/mL *in vitro*. The diversity in evaluation strategies and core parameters showed different predictive performance. The limitation of existing methods in antimicrobial activity and hit ratio and the accumulation of antimicrobial activity data provide impetus to develop a new comprehensive screening method for accelerating antimicrobial peptide discovery.

In this study, 59 physicochemical and structural parameters (also called descriptors), three strategies [decision-tree like screening model, weighted point (WP) method, and first-place amino acid preference], and four algorithms (SVM, RF, DA, and ANN) were integrated to construct a novel and comprehensive AMP mining approach, the Multiple Descriptor Multiple Strategy (MultiDS) system, for mining cryptic α-helical AMPs from global human genome database. The decision tree is a classification method by a tree structure model to solve the classification problem. It splits a dataset into subsets according to different data characteristics until the split data belongs to the same category (Quinlan, 1986; Tan et al., 2006). The WP method means a multi-parameter comprehensive rating system, in which each parameter is endowed with a weight according to its importance. Then a total point value is calculated by summing the products of every parameter and its weight. The WP method can conduct a more intensive and objective analysis of evaluating items (Thompson, 1990, 1991). Furthermore, the probability of different amino acids appearing in the N-terminal of the AMPs collected in the APD3 database^1^ was analyzed and defined as “first-place amino acid preference (FAAP).” Meanwhile, 59 parameters here, refer to the physicochemical and structural characteristics of AMP, including 28 parameters reported in the literature, 23 parameters derived by normalization or denormalization of the reported parameters, and eight parameters, proposed for the first time in this study, were selected by exploring their correlations to antibacterial activities or α-helix structures.

Subsequently, using this MultiDS system, the entire human genome, including exon and intron, was translated and scanned to find peptides ranging from 15 to 25 residues in length. From the tens of billions of possible peptides, a series of potential AMPs with MD scores were obtained. Then the antimicrobial activities of 60 peptides with top MD scores were detected against 10 kinds of pathogens *in vitro*. Furthermore, AMPs S1, S2, and S3 with top MD scores were selected to treat mouse skin wounds infected by pathogenic Gram-positive/negative bacteria and drug-resistant bacteria. The mechanism study discovered that antimicrobial action was caused by cell membrane rupture and content leakage.

## Materials and Methods

### The Construction of Multiple Descriptor Multiple Strategy Screening System

#### The Collection of Source Data

To establish a predictive and screening system for potential AMPs *in silico*, the AMPs in DBAASP (Pirtskhalava et al., 2016) (Database of Antimicrobial Activity and Structure of Peptides)^2^ were selected by the following criteria: (1) without modifications, (2) the length of 5–50 amino acid residues, (3) without D-amino acids, (4) monomeric peptide, (5) the target object was lipid bilayer, (6) the antimicrobial activity was recorded as minimum inhibitory concentration (MIC), and (7) the tested pathogens were *Escherichia coli*, *Pseudomonas aeruginosa*, and *Staphylococcus aureus*. Then the selected AMPs were submitted to the APD3 database (Antimicrobial Peptide Calculator and Predictor)^3^ for the prediction of secondary structure. The obtained peptides were divided into a series of datasets according to their antibacterial activities and pathogenic bacteria.

#### The Correlation Analyses Between 59 Parameters and the Minimum Inhibitory Concentration Values of Antimicrobial Peptides

According to literature reports, net charge, hydrophobicity, isoelectric point, amphiphilicity, and a series of physicochemical and structural parameters were commonly used to evaluate the activity of AMPs. Furthermore, the Spearman correlation coefficient of each parameter was analyzed between the parameter value and the MIC value of the datasets by using GraphPad Prism 8. The average valid correlation coefficient (AVCC) of all the Spearman coefficients of each parameter was calculated. For one given parameter, if there were both positive and negative correlations in all the Spearman correlation coefficients for each group, the majority correlation was recognized as an effective correlation, while the minority was recognized as an invalid correlation. Removing the non-correlation (*p* ≥ 0.05) and invalid correlation, the sum of all the effective correlation coefficients was divided by the number of the dataset to obtain the AVCC.

#### The Development of the Multiple Descriptor Index

The MD index was built based on the WP method, in which, the negative values of AVCC were assigned as the weight of parameters. Each parameter value corresponding to the selected AMPs was calculated and the maximum and minimum values were identified. Subsequently, the parameter value of each AMP was mapped to 0–1 using min-max normalization. The formula (1) was shown as follows:

$${\mathrm{Parameter}}^{\prime }=\frac{\text{Parameter}-{\text{Parameter}}_{m\,i\,n}}{{\text{Parameter}}_{m\,a\,x}-{\text{Parameter}}_{m\,i\,n}}$$

where the Parameter_max_ and Parameter_min_ were the maximum and minimum values of each parameter of the selected AMPs.

Then, the values of normalized parameters of a certain peptide were multiplied by their corresponding weights, and the MD’ index of each peptide was obtained by summing their products. The formula (2) was shown as follows:

$${\mathrm{MD}}^{\prime }=\sum_{i=1,\ldots ,n}^{n=45}w_{i}\,P_{i}^{\prime }$$

where P’ was the normalized parameter (parameter’), *w* was the negative value of AVCC (as the weight of each parameter), and *n* was the number of parameters.

Finally, MD’ was conducted centesimal normalization to obtain the MD index by formula (3) as follows:

MD⁢Index=100×MD′-MD′m⁢i⁢nMD′m⁢a⁢x-MD′m⁢i⁢n

In order to compare the performance between the MD index and the commonly used algorithms like SVM, RF, and DA, the AVCC for SVM, RF, and DA were calculated. The AMPs collected from the DBAASP database were submitted to the CAMP_R3_ database and scored by SVM, RF, and DA algorithms, respectively. Then, the correlation coefficients were analyzed by using the scores of each AMP calculated by each algorithm and its corresponding MIC values, and then, the average effective correlation coefficient (AVCC) value was obtained. To further verify the validity of the MD Index, the newly collected AMPs in the DBAASP database were selected to assess the correlation between the MD index and MIC by the same method as above.

#### Determination of the Cutoff Value of Parameters

According to the decision-tree-like screening model, each parameter was taken as a decision node, and the cutoff value of each parameter was set as the screening criterion for each node to evaluate whether the peptide meets the requirement. Therefore, the parameter values of the selected AMPs were calculated. Then the AVCC between parameter value and MIC was analyzed to ascertain whether the correlation was positive or negative. If the correlation between a parameter and MIC was ascertained to be negative, it indicated that the larger the parameter, the smaller the MIC, and the stronger the antibacterial activity. Subsequently, the histogram distribution of each parameter of the selected AMPs was drawn, by which the distribution range and characteristics of a specific parameter could be obtained. Based on the ascertained negative correlation, the cutoff value of the parameter was determined a higher value tending to prefer the right side in the histogram, which was more conducive for screening of AMPs with high antibacterial activity. If a positive correlation was ascertained between the parameter and MIC, the cutoff value was determined tending to prefer the left side in the histogram with a lower value. In short, different parameters had different histogram distribution characteristics, furthermore, the magnitude of correlations and “plus” or “minus” were also different between different parameters and MIC, the cutoff values of all parameters were determined manually with the orientation to maximize the possibility of screening for the AMPs with high antibacterial activity.

#### Construction of Decision-Tree Like Screening Model

In this study, a decision-tree-like screening process was used as the screening model, in which the property screening order and the cutoff value for each property were manually determined. During the construction of a decision-tree-like screening model, the parameters selected in this study and the algorithms in CAMP_R3_ were divided into three major screening steps according to whether they could be calculated offline and in different databases. Specifically, step 1 was offline calculation and screening; step 2 was online calculation and screening by DBAASP; step 3 was online filtering by the algorithms in CAMP_R3_. The detailed construction process of the decision-tree-like screening model was as follows.

Firstly, the parameters, which can be calculated offline, were connected in series to establish a step-by-step calculation procedure. In this process, each parameter was set as an independent evaluating node and the cutoff value was designed as assessment criteria. Then, the peptide to be evaluated was calculated step by step according to the evaluation process. The calculated result of the parameter was compared with the predetermined cutoff value. If the value is within the cutoff value, then move to the next parameter; if the parameter value is without the cutoff value, then stop the screening and the peptide is abandoned. Only if one peptide satisfies the cutoff value of all the parameters in the calculation, it will be output.

Secondly, the outcome peptides from Step 1 were submitted to the DBAASP website (see text footnote 2, “general property”) and evaluated by the parameters step-by-step as the calculation procedure in Step 1, and the outcome peptides that met all the preset cutoff values were submitted to the CAMP_R3_ website^4^ and calculated online by the algorithms, finally, the peptides that met all the given criteria were output.

In the step-by-step screening process, different properties could be applied in different orders, but each candidate peptide to be evaluated must simultaneously satisfy the preset cutoff value for all properties before it could be output as a qualified peptide.

#### The Analysis of First-Place Amino Acid Preference of Antimicrobial Peptides

The AMPs, with veritable α-helical structure and less than 100 amino acid residues in length deposited in the APD3 database, were selected, then statistically analyzed the frequency of the first-place (N-terminal) amino acid to determine whether there was an amino acid preference, which was defined as an FAAP.

#### The Establishment of the Multiple Descriptor Multiple Strategy System

The MultiDS method was built based on physicochemical and structural parameters, strategies, and algorithms to form an integrated system for the mining of cryptic α-helical AMPs *in silico*. The specific process of MultiDS screening was shown in section “The Establishment of MultiDS System.”

### Mining Cryptic α-Helical Antimicrobial Peptides From the Human Genome by Multiple Descriptor Multiple Strategy

The human genome sequence (2,948,583,725 bp) including 22 autosomes, two sex chromosomes, and a mitochondrion genome were downloaded from the National Center of Biotechnology Information (NCBI) website^5^. The whole-genome sequences were translated into amino acid sequences by six-frame translation from the beginning to the end, including introns and exons, coding, and non-coding regions. Subsequently, the amino acid sequences were scanned and obtained in a huge peptide library containing approximately 70 billion peptides in the length of 15–25 amino acid residues. Then, the peptides were screened and evaluated according to the MultiDS system procedure. Finally, the obtained potential AMPs were submitted to the CAMP_R3_, APD3, and DBAASP database for homology and similarity analysis to judge their novelty.

### *In vitro* Antimicrobial Activity Assay

The potential AMPs were chemically synthesized by GenScript Biotechnology Co., Ltd., Nanjing, China, with a purity of no less than 90%. The antibacterial activity measurement was modified based on the broth microdilution method (Zhong et al., 2019). A total of 10 kinds of pathogens were used, including Gram-positive bacteria, *Staphylococcus aureus* (CMCC26003), multiple-resistant *Staphylococcus aureus* (MRSA186), *Enterococcus faecium* (VRE204) (vancomycin-resistant strain), and Gram-negative bacteria, such as *Pseudomonas aeruginosa* (CMCC10104), *Escherichia coli* (CMCC44103), *Escherichia coli* (SYPB-3820) (multiple-resistant strain), *Klebsiella pneumoniae* (CMCC46117), *Acinetobacter baumannii* (ACCC11038), *Shigella dysenteriae* (CMCC(B)51105), and *Salmonella paratyphi* B (CMCC50094). Briefly, the bacteria were grown in Mueller Hinton Broth (MHB) medium (beef extract, 3 g/L, acid hydrolyzate of casein, 17.5 g/L, starch, 1.5 g/L) at 37°C to mid-log phase, and diluted to 5 × 10^5^ colony-forming units per milliliter (CFU/mL). The samples were diluted to 640 μg/ml with deionized sterile water and added to a 96-well plate that pre-contained 180 μl pathogens suspension at a gradient of 64 to 0.125 μg/ml. Thereafter, the 96-well plate was incubated at 37°C for 16–20 h, then, the absorbance at 600 nm was measured using a microplate reader (Tecan Infinite M1000 PRO). The lowest concentration, where the growth of 90% pathogens was inhibited, was taken as the minimal inhibitory concentration (MIC). The inhibition rate was calculated by using the following equation:

$$\mathrm{Inhibition}\,\mathrm{rate}=\frac{{\text{OD}}_{600\,\mathrm{nm}\,\left(\mathrm{positive}\,\mathrm{control}\right)}-{\text{OD}}_{600\,\mathrm{nm}\,\left(\mathrm{sample}\right)}}{{\text{OD}}_{600\,\mathrm{nm}\,\left(\mathrm{positive}\,\mathrm{control}\right)}-{\text{OD}}_{600\,\mathrm{nm}\,\left(\mathrm{negative}\,\mathrm{control}\right)}}\times 100\%$$

### Skin Wound Infection Healed by Antimicrobial Peptides

In order to evaluate the therapeutic effect of AMP S1, S2, and S3 *in vivo*, the full-thickness skin infection and healing experiment was carried out on mice. Briefly, adult female BALB/c mice (18–22 g) were obtained from the Laboratory Animal Center of Shenyang Pharmaceutical University and performed in compliance with the guidelines of the Institutional Animal Care and Use Committee of Shenyang Pharmaceutical University. Each mouse was operated on an 8-mm in diameter round-shape wound under surgery conditions with an intraperitoneal injection of ketamine (90 mg/kg) and xylazine (10 mg/kg). The mice were randomly divided into 10 groups, including one normal group, three model groups, three treatment groups, and three positive groups (*n* = 10 in each group), then the wounds were infected with 40 μl (1 × 10^8^ CFU/mL) of *E. coli* (CMCC44103) or drug-resistant *E. coli* (SYPB-3820) or *S. aureus* (CMCC26003), respectively, except for the normal group. Forty-eight hours later, the wounds of three model groups were treated with a sterile 0.85% saline solution. The wounds of the treatment group were treated with 100 μl S1 against *E. coli* (CMCC44103), or S2 against *E. coli* (SYPB-3820), or S3 against *S. aureus* (CMCC26003) at 16 μg/mL (8 × MIC), respectively. In the meantime, 100 μl positive drug levofloxacin was given at the concentration of 0.24 μg/ml (8 × MIC) against *E. coli* (CMCC44103), or 64 μg/ml (8 × MIC) against *E. coli* (SYPB-3820), or 0.96 μg/mL (8 × MIC) to against *S. aureus* (CMCC26003) for positive groups. All the wounds were treated twice 1 day and observed every 24 h for 14 days. On days 1, 3, 5, 7, and 10, 100 mg of treated skin tissue was removed and homogenized in 0.9 ml of sterile 0.85% saline solution, then the samples were diluted and plated onto Luria-Bertani (LB) medium. After incubation at 37°C for 20 h, the CFUs were counted to represent the bacterial number in the wounds.

### The Study of the Bactericidal Mechanism of Antimicrobial Peptides

#### Acridine Orange/Propidium Iodide Double Staining Assay

To investigate the impact of S1, S2, and S3 on the bacterial cell membrane permeability, AO/PI (Acridine Orange/Propidium Iodide) double staining assay was carried out by using Live/Dead Cell Double Staining Kit HR 0462 (Beijing Baiaolaibo Technology Co., Ltd., Beijing, China). Bacteria were cultured to the logarithmic growth stage, then washed three times in a 0.01 M phosphate-buffered saline (PBS) solution (pH 7.4) and resuspended to 1 × 10^8^ CFU/mL in PBS. Afterward, *E. coli* (CMCC44103) was treated with S1, *E. coli* (SYPB-3820) with S2, and *S. aureus* (CMCC26003) with S3, respectively, at the final concentration of 2 μg/mL (1 × MIC), then incubated at 37°C for 90 min. After centrifugation, the bacteria were subsequently treated with AO and PI at 4°C for 20 min. The samples were washed with 0.01 M PBS solution (pH 7.4), then observed and photographed by a fluorescence microscope (Olympus BX53F, Olympus, Japan). The bacteria untreated with AMPs were used as a negative control.

#### Inner Membrane Permeability Assay

The inner membrane permeability of bacteria was measured by the released activity of β-galactosidase utilizing ONPG (o-nitrophenyl-β-D-galactoside) as a substrate. *E. coli* was harvested at a logarithmic phase in the MHB medium containing 5% lactose and washed by centrifugation at 1,000 × *g* for 10 min. Afterward, the pellet was washed and resuspended to 1 × 10^8^ CFU/mL in PBS buffer. Exactly 100 μl of *E. coli* suspension and 90 μl of peptides solution (final concentration of 1 and 4 × MIC) were mixed with 10 μL of ONPG solution (30 mM) in a 96-well microtiter plate. A total of 0.5% of NaCl and 1% of Triton X-100 were served as negative and positive control, respectively. The change of absorbance at 420 nm was monitored by utilizing a microplate reader.

#### Outer Membrane Permeability Assay

The OM permeability was measured by using N-Phenyl-1-naphthylamine (NPN) fluorescent probe assay. The bacteria were harvested at the logarithmic phase and washed by centrifugation at 1,000 × *g* for 10 min and resuspended to 1 × 10^8^ CFU/mL in 5 mM 2-[4-(2-hydroxyethyl)-1-piperazinyl]ethanesulfonic acid (HEPES) buffer (pH 7.2). Then, 100 μl of bacteria suspension and 50 μl of peptides (final concentration of 1, 2, and 4 × MIC) were mixed with 50 μl of NPN (final concentration of 10 μM) in a 96-well microtiter plate. A total of 0.5% of NaCl solution and 1% of Triton X-100 solution were served as negative and positive control, respectively. The changes of fluorescence within 10 min were recorded by employing a microplate reader. The excitation and emission wavelengths were 350 and 420 nm, respectively.

#### Peptide and DNA Binding Assay

The gel retardation experiment was conducted according to the method described by Kim et al. (2018) for exploring the influence of AMPs on the bacterial genome. The bacterial genomic DNAs were extracted by using a Vazyme FastPure^®^ Bacteria DNA Isolation Mini Kit (Nanjing Vazyme Biotech Co., Ltd., Nanjing, China). A total of 10 μl genomic DNA (approximately 400 ng) was dissolved in TE buffer (10 mM Tris–HCl and 1 mM EDTA, pH 8.0) and mixed with different concentrations of peptide (final concentration of 0.5, 1, 2, 4, and 8 × MIC), and then, incubated at 37°C for 30 min. DNA treated with Penetratin (reported as cell-penetrating peptide) (de Mello et al., 2019) and DNA untreated with AMP were served as positive and negative control, respectively. The extent of DNA migration was measured by agarose gel electrophoresis using a gel imaging system (Beijing Sage Creation Science, Beijing, China).

#### Nucleic Leakage Assay

In order to study the effect of AMP on plasmalemma integrity, the leakage of nuclear acids (RNA/DNA) was determined by measuring the absorbance at 260 nm. Bacteria were harvested at the logarithmic phase by centrifugation at 3,000 × *g* for 10 min, washed three times in PBS (pH 7.4), and resuspended to an absorbance at 600 nm of 0.5. The suspensions were treated with S1, S2, or S3 at the final concentration of 1, 2, and 4 × MIC. After incubation at 37°C for 1, 2, 4, 6, and 8 h, the samples were passed through a 0.22-μm Millipore and detected the absorbance at 260 nm.

#### Scanning Electron Microscope Assay

The bacteria were cultured to the logarithmic phase, harvested, and resuspended to 10^8^ CFU/mL in PBS (pH 7.4), then treated with S1, S2, or S3 at the final concentration of 2 × MIC at 37°C for 2 h. After being washed with PBS buffer, the samples were fixed in 2.5% (v/v) glutaraldehyde solution at 4°C overnight, then dehydrated through a gradient series of ethanol (50, 70, 90, and 100%) for 15 min at each gradient, and finally dehydrated further in tert-butanol for 30 min. After coating with gold using an ion sputtering device (Hitachi E-1010, Japan), the specimens were observed using SEM (Hitachi S-3400N, Japan).

#### Transmission Electron Microscopy Assay

The logarithmic phase strains were harvested and resuspended to 10^8^ CFU/mL in PBS (pH 7.4) and treated with S1, S2, or S3 (2 × MIC) at 37°C for 2 h. Then a 10-μl bacterial suspension was dropped onto the copper grids. After sedimentation for 10 min, the liquid was absorbed away, and the samples were stained with 1% phosphotungstic acid (w/v), dried at room temperature, and examined by applying TEM (Hitachi HT7700, Japan).

### Statistical Analysis

All the results were performed by GraphPad Prism 8.0 (GraphPad Software, San Diego, CA, United States). Data represent the mean ± SD of three replicates. *, ** and *** represent *p* < 0.05, 0.01, and 0.001, respectively.

## Results

### The Establishment of the Multiple Descriptor Multiple Strategy System

**FIGURE 1:**
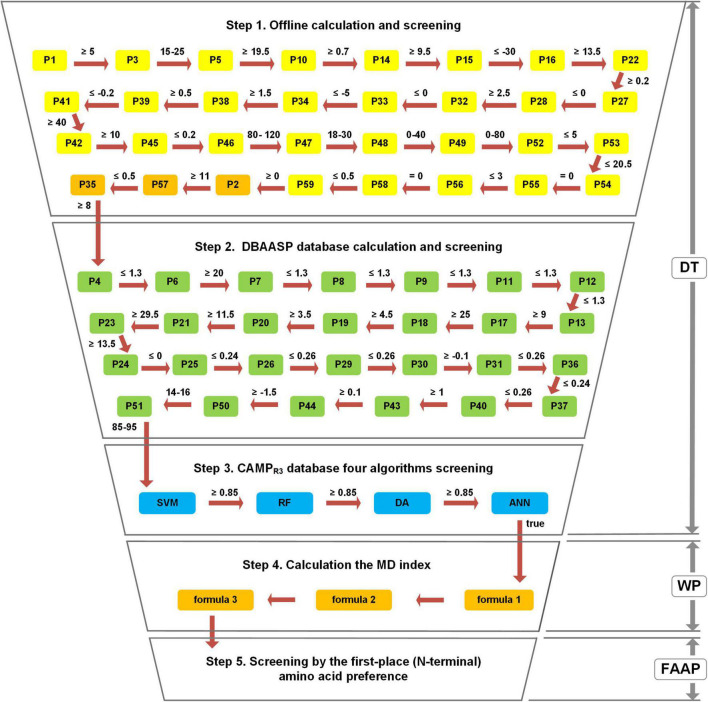
The workflow of the Multiple Descriptor Multiple Strategy (MultiDS) screening system. DT represents a decision-tree-like screening model, including three steps of offline calculation and screening, DBAASP database calculation and screening, and four algorithms screening by CAMP_R3_. WP represents the weighted point method, which was taken as the theoretical basis to construct the MD index through three calculation formulas. FAAP represents the first-place amino acid preference.

To construct a comprehensive and efficient assessment method, the MultiDS system was established based on 59 parameters, three strategies (decision-tree like screening model (DT), WP method, and FAAP), and four algorithms (SVM, RF, DA, and ANN). The specific operation process was carried out according to the following steps (Figure 1).

Step 1: Offline calculation and screening. The stepwise computation and inspection were conducted for the evaluation of putative peptides using each parameter as a decision node. If a peptide satisfies all the predetermined cutoff values, it will be output to the next step.

Step 2: Online calculation and screening by DBAASP. The outcome peptides from Step 1 were submitted to the DBAASP website (see text footnote 2, “general property”) and evaluated by 27 parameters. If a peptide satisfies all the predetermined cutoff values, it will be outputted to the next step.

Step 3: Online filtering by four algorithms (SVM, RF, DA, and ANN) in CAMP_R3_ (Waghu et al., 2016). The eligible peptides from Step 2 were submitted to the CAMP_R3_ (see text footnote 4) and calculated online by four algorithms, namely Support Vector Machines (SVM), Random Forests (RF), Discriminant Analysis (DA), and Artificial Neural Network (ANN). The only the scores given by SVM, RF, and DA were no less than 0.85 and the peptide was judged as “true” by the ANN algorithm simultaneously, can the peptide be submitted to Step 4. The above three steps constituted the decision-tree-like screening model (DT).

Step 4: Offline MD Index calculation. The peptides obtained by Step 3 were calculated according to formula 1 to get P’, and then calculated MD’ by formula 2, finally calculated MD Index by formula 3.

Step 5: Offline screening by the FAAP. The peptides that came from Step 4 were selected by FAAP strategy to filter out peptides whose first amino acid was not G, F, K, I, A, L, R, V, S, M.

Through the above five steps, the large peptide library could be rapidly compressed, which was conducive to mining new AMPs from large samples rapidly and efficiently.

#### The Collection of Antimicrobial Peptides Data and Determination of 59 Parameters

A total of 1,028 α-helical AMPs and their 3,273 data (MIC values) in the DBAASP database were collected and divided into 16 datasets according to different MIC groups and pathogenic bacteria (Supplementary Table 1). Meanwhile, a total of 59 physicochemical and structural parameters (P1–P59) were designed for the model construction, among which 28 were from literature reports, 23 were derived from the parameters in the literature by normalization or unnormalization, and eight were proposed in this study including α-helix index, normalized α-helix breaker index (Nα-B), α-helix breaker index (α-B), consecutive α-helix breakers index (Cα-B), index of two or three consecutive amino acid residues with identical charges (CC2, CC3), disulfide bond index (DSB) and α-helix Index II. The definition and parameter value calculation of 59 parameters were displayed in Supplementary Table 2.

#### Establishment and Verification of the Multiple Descriptor Index

In order to construct the MD index, 16 Spearman correlation coefficients and the AVCC between the value of 58 parameters (except P59, which was only used to distinguish helical structures) and the MIC of AMPs in 16 datasets were analyzed and listed in Supplementary Table 3. Most correlation coefficients between the MIC and parameter values of P1–P45 were valid (*p* < 0.05), and their absolute values of AVCC were greater than 0.06, displaying relatively steady and sufficient correlation strength with MIC values. Therefore, P1–P45 was selected to construct the MD Index, while, as for P46–P59, due to their own characteristics (the correlation was weak or unrobust to the MIC), they did not meet the request of the construction of the MD index, so they were not incorporated into the MD index, but designed as an independent limiting parameter in the decision-tree like screening model. For the calculation of the MD index, the maximum and minimum values of each parameter were calculated and listed in Supplementary Table 4. The MD index of 1,028 AMPs was obtained by min-max normalization for each parameter and centesimal normalization after the weighted sum. The Spearman correlation coefficients between the MD Index and MIC values of the 16 datasets were carried out. The obtained AVCC value was −0.352, which showed a consistent negative correlation (*p* < 0.05) (Supplementary Figure 1A). Moreover, the absolute value of AVCC of the MD index was larger than that of SVM, RF, and DA (−0.093, −0.206, and −0.202) (Supplementary Table 5), which indicate that the MD index has better relevance to the antibacterial activity than the commonly used algorithms.

To verify the validity of the MD Index, the latest 351 eligible AMPs with 989 MIC values deposited in the DBAASP database were selected according to the above criteria. The correlation coefficient between MD Index and MIC was carried out and the AVCC value was obtained (−0.336), which showed a consistent negative correlation (*p* < 0.05) (Supplementary Figure 1B), indicating that the MD index can be used as a valid and stable indicator for the evaluation of AMPs.

#### The Determination of the Cutoff Value of 59 Parameters

In the stepwise screening procedure of a decision-tree-like screening model, parameters P1–P58 were used as a separate indicator in the MultiDS system for screening potential AMPs, so the priority task was to determine the optimal value range (cutoff value) of each parameter. For example, the histogram distribution for P1 (net charge) of 1,028 AMPs ranged from −3 to 23, most of which were concentrated between 2 and 7 (Supplementary Figure 2). Considering that the AVCC between P1 and MIC was −0.447, which meant P1 (net charge) was negatively correlated with MIC, the cutoff value of P1 was manually determined tending to prefer a higher value of no less than 5. The cutoff value was relatively tight because the DNA sequence pool (such as the human genome used later in this study) is large enough to satisfy the screening criteria. If the object’s DNA is a small sample, moderately broad boundary values may be helpful to improve the coverage of screening targets. Therefore, the cutoff value can be designed flexibly and subjectively according to the DNA pool scale. The cutoff values of P2–P58 were manually assigned by the same method.

The P59 (α-helix II index) value of 999 AMPs with exact secondary structures (Helix, Rich, Beta, Bridge, Combine Helix, and Beta) in the APD3 database (Antimicrobial Peptide Calculator and Predictor, see text footnote 3) were calculated. The cutoff value was determined according to its histogram distribution, in which most α-helix AMPs were greater than 0, while the other four structures were less than 0 (Supplementary Figure 3). Therefore, the boundary of P59 was manually taken as no less than 0. The cutoff values of 59 parameters were listed in Supplementary Table 6.

#### Establishment of Decision-Tree Like Screening Model

The decision-tree like screening model was established by using the 59 parameters and four online algorithms as evaluating nodes and divided into three major screening steps including offline calculation and screening composed of 32 parameters, online calculation, and screening by the DBAASP database composed of 27 parameters and online filtering by four algorithms (SVM, RF, DA, and ANN) in CAMP_R3_. Through the above three major and 63 minor steps, the target peptides could be rapidly screened out for the subsequent assessment.

#### The First-Place Amino Acid Preference of Antimicrobial Peptides

**FIGURE 2:**
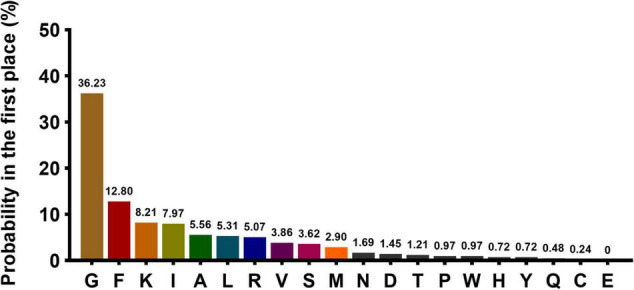
The probability of the 20 amino acids present at the N terminal. The *X*-axis represents 20 amino acids, and *Y*-axis represents the probability of each amino acid occurring in the first place at the N terminal.

A total of 414 AMPs that met the criteria were selected for the first amino acid preference analysis. Statistical analyses showed that 10 kinds of amino acids (G, F, K, I, A, L, R, V, S, and M) presented with high frequency (sum up to 91.5%) in the first-place of AMPs. The top 10 amino acid were listed in descending order: Glycine (G, 36.23%), phenylalanine (F, 12.80%), lysine (K, 8.21%), isoleucine (I, 7.97%), alanine (A, 5.56%), leucine (L, 5.31%), arginine (R, 5.07%), valine (V, 3.86%), serine (S,3.62%), and methionine (M, 2.90%) (Figure 2). The rule of FAAP was integrated into the MultiDS system as a unique strategy for narrowing the range of potential antimicrobial peptides.

### Mining of α-Helical Antimicrobial Peptides From the Human Genome by the Multiple Descriptor Multiple Strategy System

**FIGURE 3:**
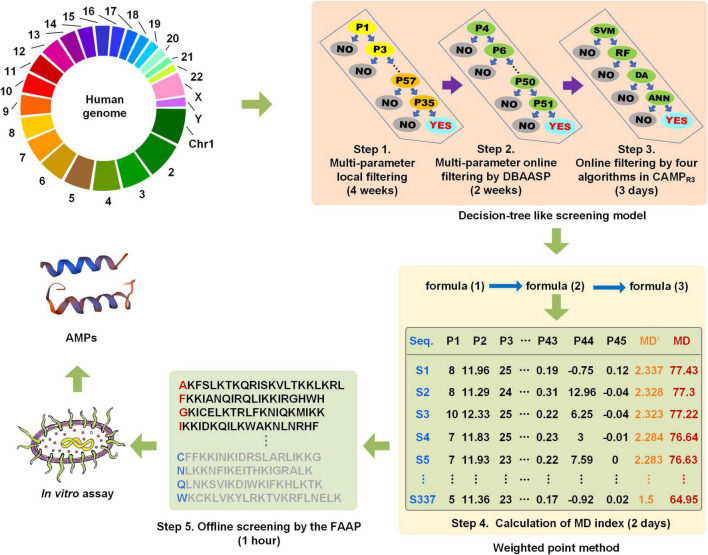
The schematic diagram of the screening of cryptic α-helical antimicrobial peptides (AMPs) with the MultiDS system.

The whole human genome (including the coding region and non-coding region) was performed the six reading frames translation, and then a total of approximately 70 billion peptides with 15–25 amino acid residues were generated and screened following the operation procedure in Figure 3 in approximately 7 weeks. Subsequently, a series of peptides with the MD index that met with all the screening criteria of the MultiDS system were exported. The 337 peptides with MD scores greater than 65 were submitted to the CAMP_R3_ database for homology analysis. Generally, the *E*-value less than 10^–5^ was considered a high homology. As for the 337 peptides, the *E*-values were in the range of 0.008–9.6, except for the peptide S105 with high homology to the Human KS-27 sequence, indicating the 336 potential AMPs had a low homology with the existing AMPs in the CAMP_R3_. Similarly, S1–S337 were also put into the APD3 database for similarity analysis, and the similarity percentage ranged from 36 to 52.17% except for S105. Likewise, there was no similar AMP to S1–S337 except for S105 in the DBAASP database. The results validated that the 337 peptides screened from the human genome by the MultiDS system were novel sequences.

### Antimicrobial Activity Assay of the Potential Antimicrobial Peptides

Considering the cost of solid-phase synthesis, a library composed of 60 peptides (S1–S60), with the top MD index score (Supplementary Table 7), were synthesized and assessed their antimicrobial activities against 10 kinds of clinically relevant pathogens *in vitro*, including Gram-positive bacteria *S. aureus* (CMCC26003), multiple-resistant *S. aureus* (MRSA186), *E. faecium* (VRE204) (vancomycin-resistant strain), and Gram-negative bacteria *P. aeruginosa* (CMCC10104), *E. coli* (CMCC44103), *E. coli* (SYPB-3820) (multiple-resistant strain), *K. pneumoniae* (CMCC46117), *A. baumannii* (ACCC11038), *S. dysenteriae* (CMCC(B)51105), and *S. paratyphi* B (CMCC50094). The results showed that 59 entities exhibited antimicrobial activities (MIC ≤ 64 μg/mL), validating the MultiDS screening system had a 98.3% hit rate for the prediction of encrypted AMPs. Fifty-four entities displayed the MIC value of no more than 8 μg/mL, showing a 90% hit rate for moderate activity AMPs, and 24 entities possessed MIC values as low as 2 μg/ml, indicating a 40% hit rate for high antimicrobial activity AMPs (Table 1). The results validated the MultiDS system was a more comprehensive and efficient approach to screening AMPs with a high hit ratio and high antimicrobial activity (MIC ≤ 2 μg/ml).

**TABLE 1:** The antimicrobial activities of S1–S60.

Name	MD	1	2	3	4	5	6	7	8	9	10
S1	77.43	2	16	2	8	2	4	2	4	4	4
S2	77.30	2	16	2	8	2	4	2	2	2	4
S3	77.22	2	16	2	2	2	2	2	2	2	2
S4	76.64	32	>64	64	64	16	8	16	8	16	32
S5	76.63	4	>64	8	64	8	8	8	4	4	8
S6	76.60	4	>64	8	64	8	16	16	4	4	8
S7	76.46	32	>64	64	>64	64	64	64	8	16	32
S8	76.39	4	>64	16	64	16	16	16	2	2	2
S9	76.39	16	64	16	64	32	64	64	16	16	32
S10	76.22	8	>64	32	64	16	32	32	4	4	8
S11	76.16	8	32	8	16	8	16	8	16	4	16
S12	76.15	4	32	4	16	2	4	4	4	4	4
S13	76.06	4	64	16	16	4	8	4	4	4	8
S14	75.96	8	>64	32	64	8	16	16	8	4	32
S15	75.85	2	64	4	32	4	8	4	4	4	8
S16	75.72	4	16	4	8	4	8	4	4	4	8
S17	75.61	4	16	4	8	4	4	8	8	4	8
S18	75.54	32	>64	16	32	64	8	64	32	16	32
S19	75.33	4	>64	4	16	4	4	4	8	2	8
S20	75.26	8	>64	16	32	4	8	4	8	4	16
S21	75.22	4	64	16	16	4	8	8	8	4	16
S22	75.18	4	>64	16	16	4	8	8	8	4	16
S23	74.99	8	64	32	16	8	16	8	8	4	16
S24	74.74	4	8	8	8	4	8	4	8	2	4
S25	74.72	8	>64	64	32	8	16	8	8	2	16
S26	74.71	4	64	8	32	8	16	16	16	2	8
S27	74.70	4	16	4	8	8	8	8	16	4	8
S28	74.62	>64	>64	>64	>64	>64	>64	>64	>64	64	>64
S29	74.61	8	32	8	16	16	16	16	8	4	8
S30	74.44	4	16	4	8	8	8	8	8	2	4
S31	74.40	8	32	8	64	16	>64	16	16	2	2
S32	74.35	4	64	8	>64	8	>64	32	8	2	4
S33	74.34	4	32	16	16	8	8	4	8	4	2
S34	74.34	8	64	16	64	8	8	8	16	2	2
S35	74.27	16	>64	32	64	8	>64	8	16	4	4
S36	74.26	4	4	4	8	4	>64	4	8	2	2
S37	74.26	8	>64	32	64	32	32	>64	>64	64	64
S38	74.25	4	>64	32	32	8	8	4	4	2	4
S39	74.17	8	64	32	16	8	4	2	8	2	16
S40	74.13	4	32	4	8	8	4	2	8	2	8
S41	74.08	8	>64	16	16	8	4	4	8	2	4
S42	74.06	>64	>64	>64	>64	>64	64	>64	>64	16	>64
S43	74.03	16	>64	64	16	8	4	4	4	4	8
S44	74.01	8	>64	8	8	8	8	4	8	2	4
S45	74.00	32	32	16	32	16	16	32	16	16	32
S46	73.91	8	16	4	8	8	2	8	4	2	8
S47	73.87	16	64	32	32	16	16	8	8	4	16
S48	73.86	32	>64	>64	>64	16	8	8	8	4	64
S49	73.84	4	>64	>64	>64	32	32	16	8	32	>64
S50	73.80	32	>64	>64	32	16	4	4	4	4	16
S51	73.79	32	>64	32	64	32	32	32	16	16	64
S52	73.78	32	>64	32	64	32	16	16	16	8	32
S53	73.75	8	>64	16	8	8	8	4	4	4	8
S54	73.65	8	>64	32	16	8	4	4	4	4	16
S55	73.64	>64	>64	>64	>64	>64	>64	>64	>64	>64	>64
S56	73.63	8	>64	64	32	8	8	4	8	4	16
S57	73.61	4	16	4	8	8	8	8	4	4	8
S58	73.45	16	>64	64	32	16	16	16	8	8	16
S59	73.36	4	16	4	8	4	4	4	4	2	8
S60	73.35	4	64	4	16	4	2	4	4	2	16
Levofloxacin	0.12	–	–	0.5	0.03	0.03	8	0.06	0.06	0.03
Vancomycin	–	1	32	–	–	–	–	–	–	–

*The color from dark red to dark blue represents the MIC from 2 to >64 μg/ml. The numbers in the first line: 1. S. aureus (CMCC26003), 2. multiple-resistant S. aureus (MRSA186), 3. E. faecium (VRE204) (vancomycin-resistant strain), 4. P. aeruginosa (CMCC10104), 5. E. coli (CMCC44103), 6. K. pneumoniae (CMCC46117), 7. E. coli (SYPB-3820) (multiple-resistant strain), 8. A. baumannii (ACCC11038), 9. S. dysenteriae (CMCC(B)51105), 10. S. paratyphi B (CMCC50094). Levofloxacin and vancomycin were used as control.*

### Therapeutic Efficacy of S1, S2, and S3 in Mice Models of Skin Wound Infection

**FIGURE 4:**
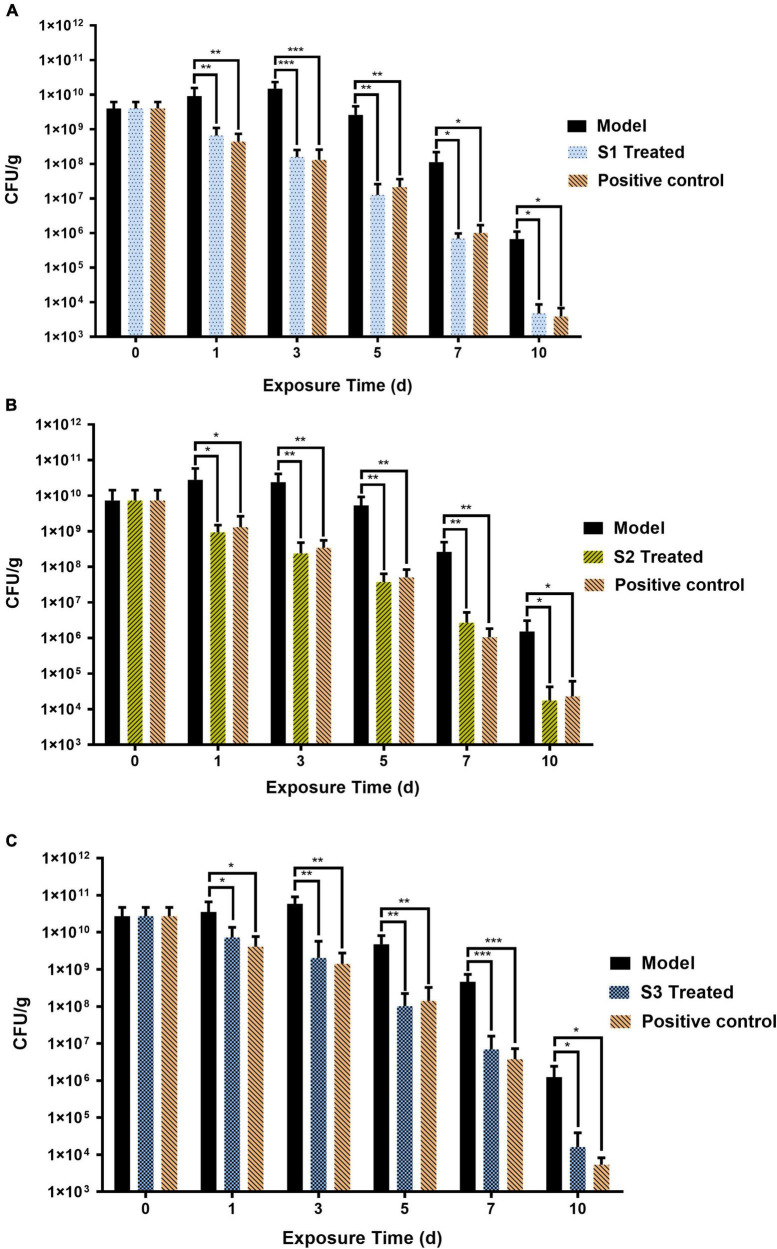
The therapeutic effects of S1, S2, and S3 in mice skin wound infection. Skin wound bacterial burden on days 0, 1, 3, 5, 7, and 10 in mice skin infected by **(A)** *E. coli* (CMCC44103), **(B)** *E. coli* (SYPB-3820), and **(C)** *S. aureus* (CMCC26003). *, **, *** represent *p* < 0.05, *p* < 0.01, and *p* < 0.001, respectively.

In order to evaluate the anti-infection therapeutic efficacy of the AMPs obtained from the MultiDS system, a mouse skin wound infection model infected with *E. coli* (CMCC44103), *E. coli* (SYPB-3820) (drug-resistance bacteria), and *S. aureus* (CMCC26003) was established. AMPs S1, S2, and S3 were selected as the representative samples to heal the wounds, and compared to the positive drug levofloxacin. As shown in Figure 4A, the initial bacterial burden in the wound infected with *E. coli* (CMCC44103) was (3.98 ± 2.15) × 10^9^ CFU/g, after treatment of S1 or levofloxacin, the number decreased to (4.72 ± 3.94) × 10^3^ CFU/g and (3.91 ± 2.87) × 10^3^ CFU/g, respectively, at day 10, which significantly decreased the bacterial load by six orders of magnitude. A similar phenomenon was observed in the other two bacteria. The initial bacterial burden in the wound infected with *E. coli* (SYPB-3820) was (7.33 ± 7) × 10^9^ CFU/g, and the number decreased to (1.75 ± 2.48) × 10^4^ CFU/g and (2.29 ± 3.78) × 10^4^ CFU/g after treatment with S2 and levofloxacin, respectively, 10 days later (Figure 4B), which reduced the bacterial counts by five orders of magnitude. The initial bacterial burden in the wound infected with *S. aureus* (CMCC26003) was (2.73 ± 1.96) × 10^10^ CFU/g, and the number decreased to (1.61 ± 2.30) × 10^4^ CFU/g and (5.37 ± 2.89) × 10^3^ CFU/g after treatment with S3 and levofloxacin, respectively, (Figure 4C), which significantly reduced by six and seven orders of magnitude. Of note, the wound bacterial remnants all decreased below 10^5^ CFU/g after 10 days, significantly lower than the untreated group, which indicated that S1, S2, and S3 could significantly kill the bacteria and promote wound healing, and exhibited comparable potency to levofloxacin in the skin wound infection therapy.

**FIGURE 5:**
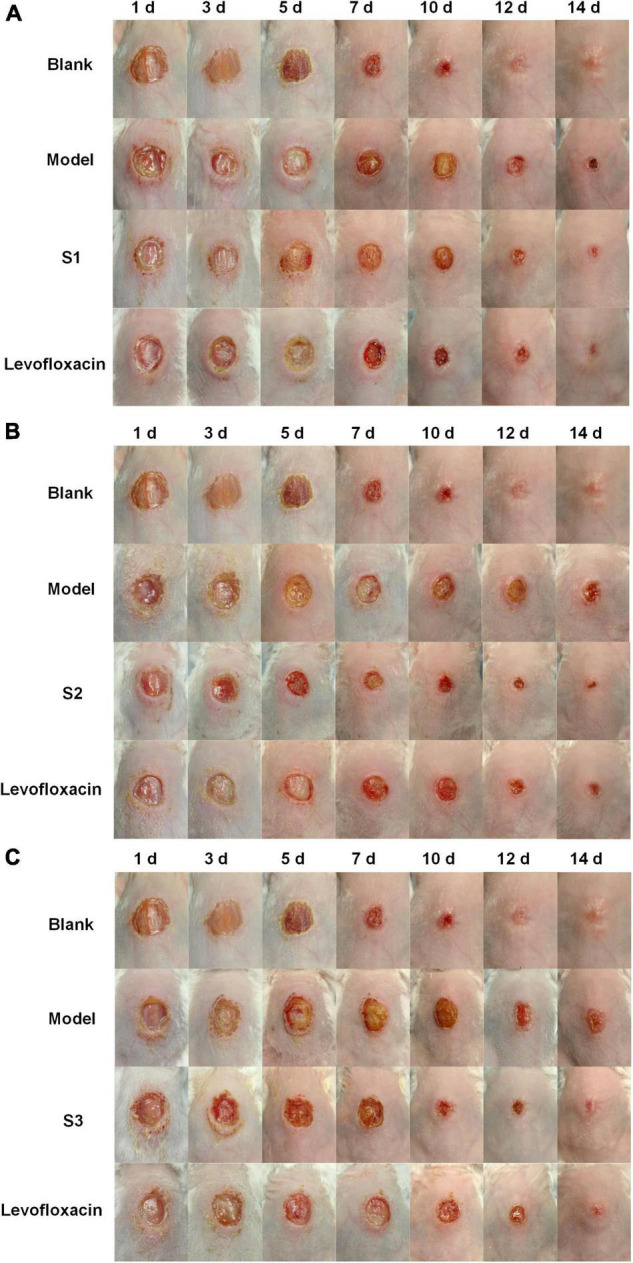
The effects of S1, S2, and S3 on promoting wound healing. The healing of wounds on days 1, 3, 5, 7, 10, 12, and 14 in mice skin infected by **(A)** *E. coli* (CMCC44103), **(B)** *E. coli* (SYPB-3820), and **(C)** *S. aureus* (CMCC26003).

On the other hand, the effects of S1, S2, and S3 on promoting skin wound healing against pathogen infection were shown in Figure 5. On day 1 of treatment, the wounds were all severely infected; on days 3–5, the redness and swelling of wounds in the S1, S2, S3, and levofloxacin treated groups began to decrease significantly, while the model groups (untreated with any drugs) had no visible improvement. On day 7, the redness and swelling of the wounds subsided and began to form scabs in the treatment groups, while the model groups recovered very slowly. On day 14, the wounds of the treated groups were basically completely healed, obviously better than the model groups. The blank group (wounds that were not infected by any bacteria and were not treated by any drugs) were also healed completely. In conclusion, S1, S2, and S3 can significantly decrease the bacterial burden and promote skin wound healing, and the antibacterial effect was comparable to levofloxacin, which indicated that S1, S2, and S3 have potential as new antibacterial agents.

### Mechanism Study

To investigate the bactericidal mechanism of the AMPs obtained by MultiDS screening, S1, S2, and S3 were selected as the representatives to act against *E. coli* (CMCC44103), *E. coli* (SYPB-3820), and *S. aureus* (CMCC26003), respectively.

#### Acridine Orange/Propidium Iodide Double Staining Assay

**FIGURE 6:**
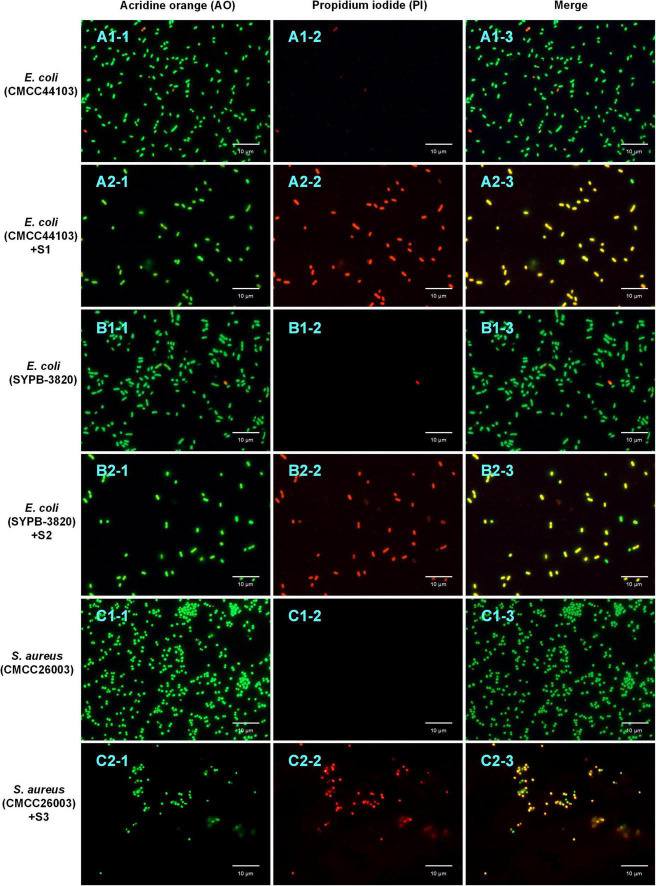
AO/PI double staining assay. The bacteria stained with AO **(A1-1,A2-1,B1-1,B2-1,C1-1,C2-1)** and PI **(A1-2,A2-2,B1-2,B2-2,C1-2,C2-2)** were photographed under the excitation of 488 and 535 nm, respectively; the merge **(A1-3,A2-3,B1-3,B2-3,C1-3,C2-3)** was fusion-fluorescence imaging under the excitation of 488 and 535 nm. The scale bar is 10 μm.

Acridine Orange/Propidium Iodide double staining was observed under fluorescence microscopy. As shown in Figures 6A1-1,A2-1,B1-1,B2-1,C1-1,C2-1, all the bacteria (treated or untreated with AMPs) showed green fluorescence under the excitation of 488 nm, indicating that AO can penetrate all cell membranes. Compared to the control groups (Figures 6A1-2,B1-2,C1-2) did not show orange fluorescence, the treated groups (Figures 6A2-2,B2-2,C2-2) manifested a bright orange fluorescence under the excitation of 535 nm, indicating that PI had entered bacteria and combined with DNA. The merged microscopic images (Figures 6A1-3,A2-3,B1-3,B2-3,C1-3,C2-3) showed that the untreated control strain cells appeared green and the AMP treated cells appeared yellow, indicating the cell membrane of control strain were intact and that of AMP treated strain were damaged. Since PI cannot enter the intact cell membranes but only pass through the damaged membranes, it is speculated that S1, S2, and S3 destroyed the bacterial cell membranes and enhanced the cell permeability.

#### Inner Membrane Permeability Assay

**FIGURE 7:**
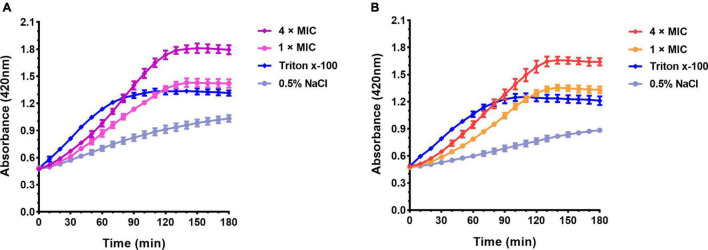
Absorbance curve at 420 nm of ONPG hydrolyzed by β-galactosidase. **(A)** *E. coli* (CMCC44103) was treated with S1, **(B)** *E. coli* (SYPB-3820) was treated with S2.

To investigate the destructive effect of peptides on the cell cytoplasmic membrane, an inner membrane (IM) permeability assay was performed. If a peptide destroyed the cytoplasmic membrane and induced cellular permeabilization, the extracellular substrate ONPG would enter the cell and be degraded into o-nitrophenol by the β-galactosidase from the inner membrane, then the product o-nitrophenol could be detected at 420 nm. As shown in Figures 7A,B, the absorbance at 420 nm increased in groups S1 and S2 at the concentration of 1 × MIC and 4 × MIC faster than that of the negative control (0.5% NaCl), indicating that S1 and S2 could improve the membrane permeability of *E. coli* (CMCC44103) and *E. coli* (SYPB-3820), respectively. The absorbance of 4 × MIC was higher than that of 1 × MIC, indicating the destructive effect of S1 and S2 was concentration-dependent. The absorbance of the positive control (1% Triton X-100) increased rapidly in the first hour, while that of the negative control (0.5% NaCl) raised slowly. The reason may be attributed to ONPG entering intact *E. coli* cells slowly, but penetrating into the damaged cell quickly. As for *S. aureus* (CMCC26003), no change in absorbance was detected at 420 nm whatever treated with S3, 1% Triton X-100 or 0.5% NaCl, this may be related to the low or no expression of β-galactosidase in *S. aureus*.

#### Outer Membrane Permeability Assay

**FIGURE 8:**
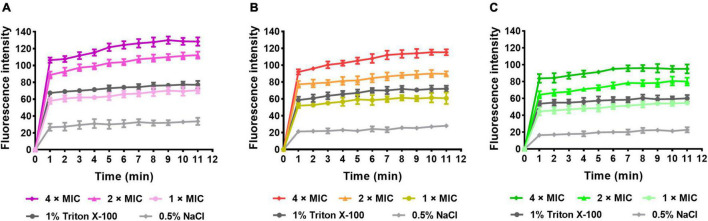
NPN uptake assay. **(A)** *E. coli* (CMCC44103) was treated with S1, **(B)** *E. coli* (SYPB-3820) treated with S2, **(C)** *S. aureus* (CMCC26003) treated with S3.

To assess the OM permeabilization by AMPs, the NPN assay was carried out. The hydrophobic probe NPN can bind to the hydrophobic part of the bacterial outer membrane and produces a strong fluorescence at 420 nm in hydrophobic environments. As observed in Figure 8, the fluorescence of group S1 (Figure 8A), S2 (Figure 8B), S3 (Figure 8C), and positive control (1% Triton X-100) increased rapidly within the first minute and slowly thereafter, but all were higher than the negative control (0.5% NaCl). Meanwhile, the excitation levels showed concentration dependence. The results indicated that S1, S2, and S3 disrupted the outer membrane of the bacteria and promoted permeability.

#### Peptide and DNA Binding Assay

**FIGURE 9:**
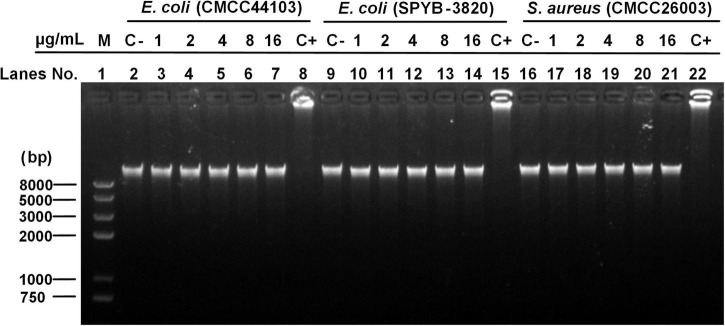
DNA binding assay. Line 1 (M) was the Trans2K Plus II DNA marker; Lines 2, 9, and 16 (C–, negative control) were the genome DNA untreated with AMP; Lines 8, 15, and 22 (C+, positive control) were the genome DNA treated with Penetratin; Lines 3–7, 10–14, and 17–21 were the DNA of *E. coli* (CMCC44103), *E. coli* (SYPB-3820), and *S. aureus* (CMCC26003) treated with S1, S2, and S3, respectively, under a concentration gradient of 1, 2, 4, 8, and 16 μg/ml.

Peptide and DNA binding assay was tested to assess peptide-induced inhibition of genomic DNA migration. As shown in Figure 9, after incubation of S1 with the DNA of *E. coli* (CMCC44103) (lanes 3–7), S2 with *E. coli* (SYPB-3820) (lanes 10–14), S3 with *S. aureus* (CMCC26003) (lanes 17–21) and negative control (without peptide incubation, lanes 2, 9, 16), all the DNA migrations were obvious without blocking or trailing phenomenon, and no DNA remained in the sample ports, which meant S1, S2, and S3 did not bind to bacterial genomic DNA. On the contrary, the positive group (lanes 8, 15, 22) showed DNA aggregated in the loading holes without migration, indicating that the positive control, Penetratin, bound with the bacterial genomic DNA and blocked the migration as the precious report (de Mello et al., 2019). The experiment proved that S1, S2, and S3 did not exert antibacterial effects through interference with genomic DNA.

#### Nucleic Acid Leakage Assay

**FIGURE 10:**
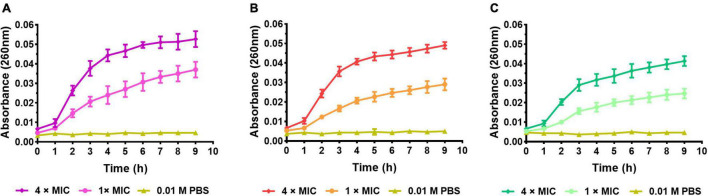
Nucleic acid leakage assay. **(A)** *E. coli* (CMCC44103) treated with S1, **(B)** *E. coli* (SYPB-3820) treated with S2, **(C)** *S. aureus* (CMCC26003) treated with S3.

The nucleic acid leakage assay was conducted to investigate the effect of peptides on bacterial cell membranes integrity by detecting the UV absorption of intracellular nucleic acid leakage at 260 nm. As shown in Figure 10, the absorbance of all the negative control was close to 0, indicating the bacterial cell membrane was intact. After being treated with S1, S2, and S3, the absorbance at 260 nm was obviously changed with the time course. The absorbance of 4 × MIC groups rose rapidly in the first 3 h, then slowed down and approached a plateau after 4 h. It meant the high concentration of S1, S2, and S3 could cause rapid cell membrane disintegration and produce cellular content leakage. Correspondingly, although the absorbance of 1 × MIC groups was lower than that of 4 × MIC groups, it was still higher than that of the negative groups, indicating S1, S2, and S3 could still damage the cell membrane at the concentration of 1 × MIC.

#### Scanning Electron Microscope Observation

**FIGURE 11:**
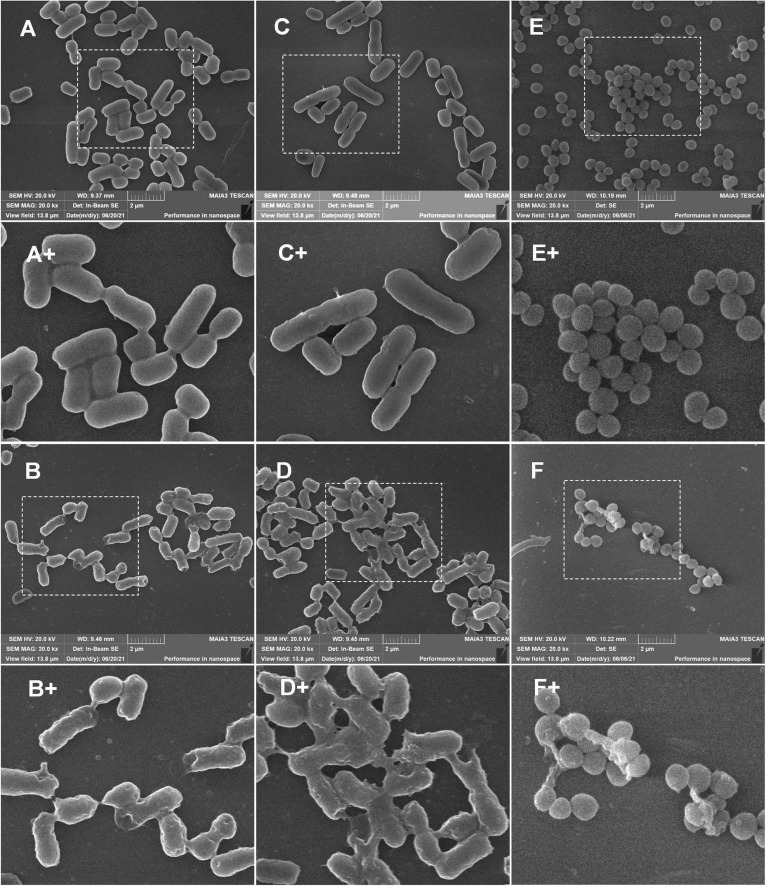
SEM observation of bacterial morphology. **(A,C,E)** Was untreated *E. coli* (CMCC44103), *E. coli* (SYPB-3820), and *S. aureus* (CMCC26003). **(A+,C+,E+)** Were the partial enlarged details of **(A,C,E)**, respectively. **(B,D,F)** Were *E. coli* (CMCC44103), *E. coli* (SYPB-3820), and *S. aureus* (CMCC26003) treated with S1, S2, and S3, respectively. **(B+,D+,F+)** Were the partial enlarged details of **(B,D,F)**, respectively.

The scanning electron microscope (SEM) was used to observe the damage to the cell membrane. As shown in Figures 11A,C,E,A+,C+,E+ (the partial enlarged detail), *E. coli* (CMCC44103), *E. coli* (SYPB-3820) and *S. aureus* (CMCC26003) untreated with peptides showed bright and smooth surface. After being treated with peptides at 2 × MIC for 2 h, the morphology was irregular and dented, and the vast majority of the bacterial surface became rough, wrinkled, and was seriously damaged. The cellular membrane breakage and disintegration further induced content leakage and bacteria adhesion (Figures 11B,D,F,B+,D+,F+).

#### Transmission Electron Microscopy Observation

**FIGURE 12:**
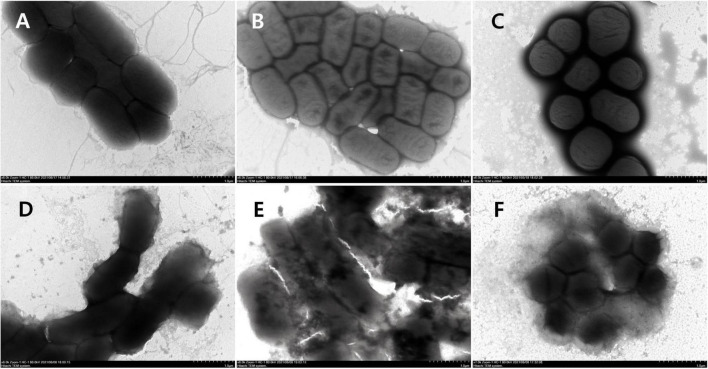
TEM observation of bacterial morphology. **(A)** *E. coli* (CMCC44103), **(B)** *E. coli* (SYPB-3820), **(C)** *S. aureus* (CMCC26003), **(D)** *E. coli* (CMCC44103) treated with S1, **(E)** *E. coli* (SYPB-3820) treated with S2, **(F)** *S. aureus* (CMCC26003) treated with S3.

The morphological and intracellular alterations of bacteria treated with AMPs were observed by using transmission electron microscopy (TEM). As shown in Figures 12A–C, the cellular surface of control bacteria without peptide treatment was intact and smooth. On the contrary, the surface morphology of *E. coli* (CMCC44103) treated with S1, *E. coli* (SYPB-3820) with S2, and *S. aureus* (CMCC26003) with S3 had dramatic changes. The cell boundary became irregular and blurred, and the collapse of the cell membrane resulted in the leakage of cell contents and dispersion of cell debris (Figures 12D–F).

## Discussion

With the rapid development of computer technology and bioinformatics, the discovery of novel AMPs by computer-assisted screening has been widely used, and more than 30 prediction methods based on diverse data quality, various core algorithms, and evaluation strategies have been put forward (Xu et al., 2021). However, most of the current studies mainly focus on the mining of AMPs from proteome or open reading frames of genomic sequence. Few studies have explored the whole genome, including introns and exons as screening targets. In order to accelerate the discovery of novel and effective AMPs, this work firstly constructed a high-through versatile AMP screening system from three aspects of parameters, strategies, and algorithms, and then comprehensively screened and systematically evaluated the huge peptide library derived from the six-frame translation of the global human genome to predict α-helical AMPs *in silico*.

Among the 59 parameters, 28 parameters were derived from literature, 23 parameters were obtained after normalization or unnormalization, and eight parameters were created for the first time in this work. When using multiple parameters, the combination of reasonable strategies can improve the screening efficiency and hit ratio, otherwise, it will produce huge calculations and increase the difficulty of screening. The decision-tree-like screening model is the key to improving the efficiency of screening AMPs from huge DNA or amino acid sequences. It is suitable to realize step-by-step screening through binary classification and to improve the hit rate by multiple parameters filtering. On the other hand, the multiple parameters filtering system is beneficial for the evaluation of peptides from multiple facets. The MD index, as the core part of the MultiDS system, is proposed based on the WP method, which contributed to the synthetic assessment of AMPs by various parameters from a holistic perspective. Through the evaluation of the MD index, the peptides are assessed in terms of getting a comprehensive score based on the performance of their parameter values and weights, thereby avoiding some parameters from being too influential to conceal other weak parameters. The frequency of the amino acid that appeared in the first place (N-terminal) showed obvious preference. Some kinds of amino acids appeared more frequently, just like “hot-spots,” showing this type of amino acid may be associated with the activity of peptide. Hence, the FAAP was used in this work to shrink the AMPs pool in the last step to improve the hit ratio.

In the screening process, the boundary value of each parameter is not fixed and can be adjusted according to the amount of data to be screened. If the object is large samples, tighter boundary values can improve the screening efficiency; if the object is small samples such as microbial genome, moderately broad boundary values may be helpful to improve the coverage of screening targets. Therefore, the MultiDS system was an open screening and evaluation system, and the parameter value range can be adjusted according to the capacity of the screening sample.

Although several computational methods have been taken to screen AMPs from various samples (Li et al., 2018; Porto et al., 2018; Tan et al., 2019; Sharma et al., 2021), the systematic screening of encrypted AMP from the global human genome has not been performed. In this study, 337 peptides with 15–25 amino acid residues were screened out from the entire human genome including intron and exon with a high MD score (≥65) by the MultiDS system, and 60 entities were synthesized to test the antimicrobial activities. As the results in Table 1 states, 98.3% of entities showed activity against pathogen (MIC ≤ 64 μg/mL), 90% of entities have moderate activity with a MIC of 8 μg/mL, and 40% of entities have strong activity with a MIC of 2 μg/mL, which is superior to the recently reported computational methods. Liu et al. (2018) established an activity prediction method based on the predicted 3D descriptors of AMP, in which, the antibacterial effect of the novel AMPs designed by this method was from 32 to 512 μg/ml. Kavousi et al. (2020) utilized the ^13^C-NMR spectral of amino acid combined with the physicochemical properties of AMPs, such as amino acid acidity and basicity, size, charged percentages, and so on, to establish a computational approach to predict AMPs, and the result showed a 95% accuracy but no detailed antimicrobial activity released. The artificial intelligence method reported by Torres et al. (2021) to predict AMP from human proteome showed a 63.6% hit rate, and 55 synthesized representative peptides showed MIC values from 0.39 to 128 μg/mL against pathogens. By contrast, the MultiDS system allows for the high-through mining of novel AMPs from global genome sequences with a 98.3% hit ratio. As expected, the mechanism study verified the AMPs destroyed the integrity of the cell membrane and resulted in the leakage of bacterial contents. Furthermore, the mouse model of skin wound infection revealed that S1, S2, and S3 had a significant effect on promoting skin wound healing caused by Gram-negative bacteria (*E. coli* and resistant *E. coli*) and Gram-positive bacteria (*S. aureus*), and their effects were comparable to that of the positive control drug levofloxacin.

The MultiDS system opens up a new route for the rapid discovery of candidate antibiotics from the global genome sequence, and also provides a new strategy for improving the hit rate with better predictive performance. The identification of correlation between parameters and MIC values quantitatively provides the effects of different parameters on the antimicrobial activity, which brings a new perspective for optimizing the existing AMPs to improve their antibacterial activity through comprehensive system analyses. Of course, there is considerable room for improvement in optimizing the screening system because the MultiDS system is a primary and open system at this stage. With the continuous development in the AMPs prediction field and accumulation of experimental data, the MultiDS system will be further refined and provide valuable screening methods to accelerate the discovery of AMPs.

## Data Availability Statement

The raw data supporting the conclusions of this article will be made available by the authors, without undue reservation.

## Ethics Statement

The animal study was reviewed and approved by the Institutional Animal Care and Use Committee of Shenyang Pharmaceutical University.

## Author Contributions

LL collected the data, designed and performed the experiments, and wrote the manuscript. CW designed and performed the experiments and wrote the manuscript. MZ and YW assisted in the antibacterial activity assay. ZZ assisted in skin wound infection. YZ designed and supervised the experiments and revised the manuscript. All authors contributed to the article and approved the submitted version.

## Conflict of Interest

The authors declare that the research was conducted in the absence of any commercial or financial relationships that could be construed as a potential conflict of interest.

## Publisher’s Note

All claims expressed in this article are solely those of the authors and do not necessarily represent those of their affiliated organizations, or those of the publisher, the editors and the reviewers. Any product that may be evaluated in this article, or claim that may be made by its manufacturer, is not guaranteed or endorsed by the publisher.

## Footnotes

1
https://aps.unmc.edu/database

2
https://dbaasp.org/

3
https://aps.unmc.edu/prediction

4
http://www.camp.bicnirrh.res.in/predict/

5
https://www.ncbi.nlm.nih.gov/genome?LinkName=assembly_genome&from_uid=2334371
