# Supplementary material for: An Efficient Evaluation System Accelerates α-Helical Antimicrobial Peptide Discovery and Its Application to Global Human Genome Mining
Source: Front Microbiol. 2022 Apr 25;13:870361. doi: 10.3389/fmicb.2022.870361 (PMC9083330; doi:10.3389/fmicb.2022.870361)
Supplement: Supplementary file 1 [file Table_1.DOCX]

Supplementary Material

# Supplementary Figures and Tables

## Supplementary Figures


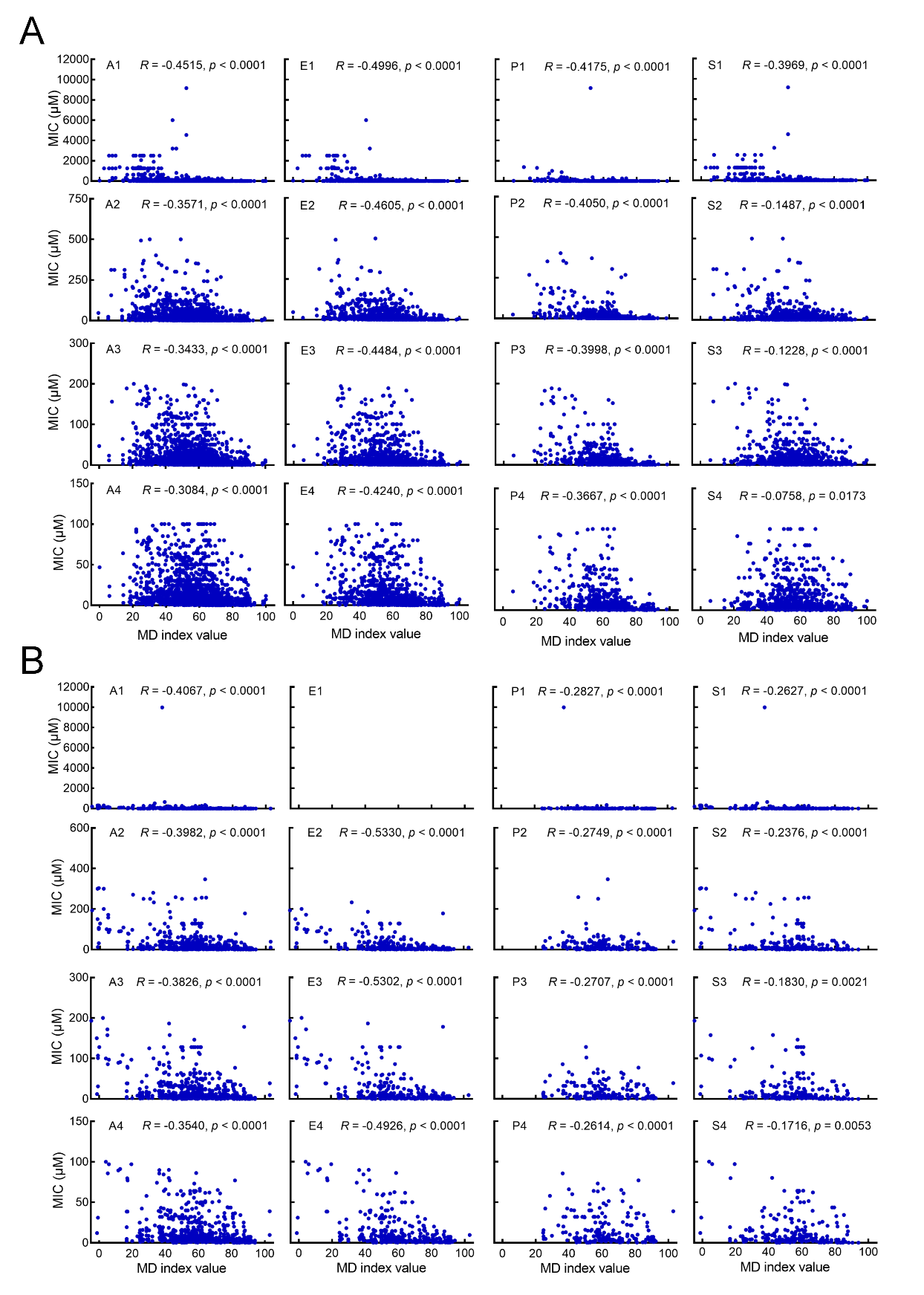


**Supplementary Figure S1** Spearman correlations between MD index values and MIC datasets. (A) The Spearman correlation analysis for MD Index of 1028 AMPs and 16 MIC datasets. (B) The Spearman correlation analysis for MD Index of 351 AMPs and 16 MIC datasets. *p* value indicates the level of significance.


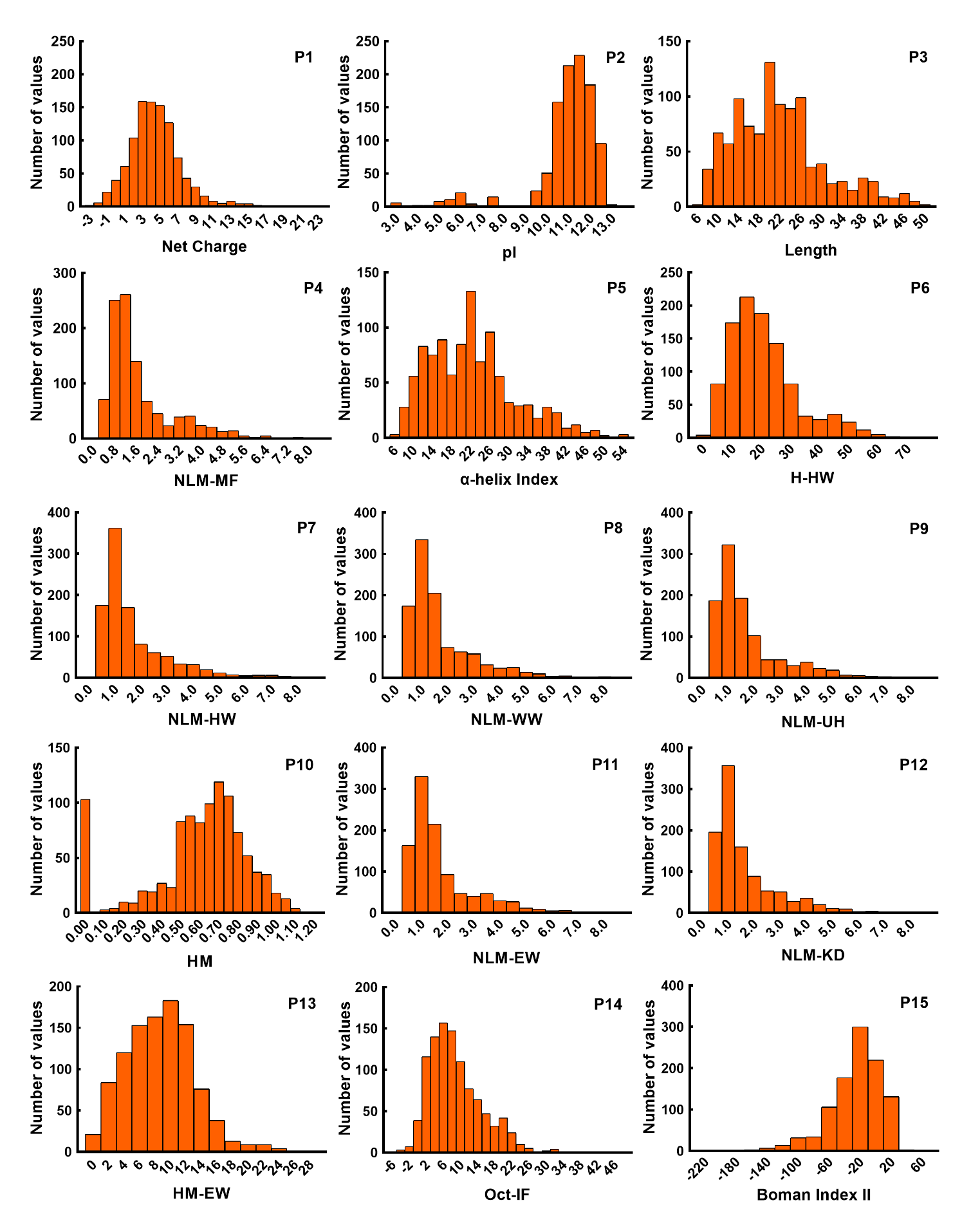


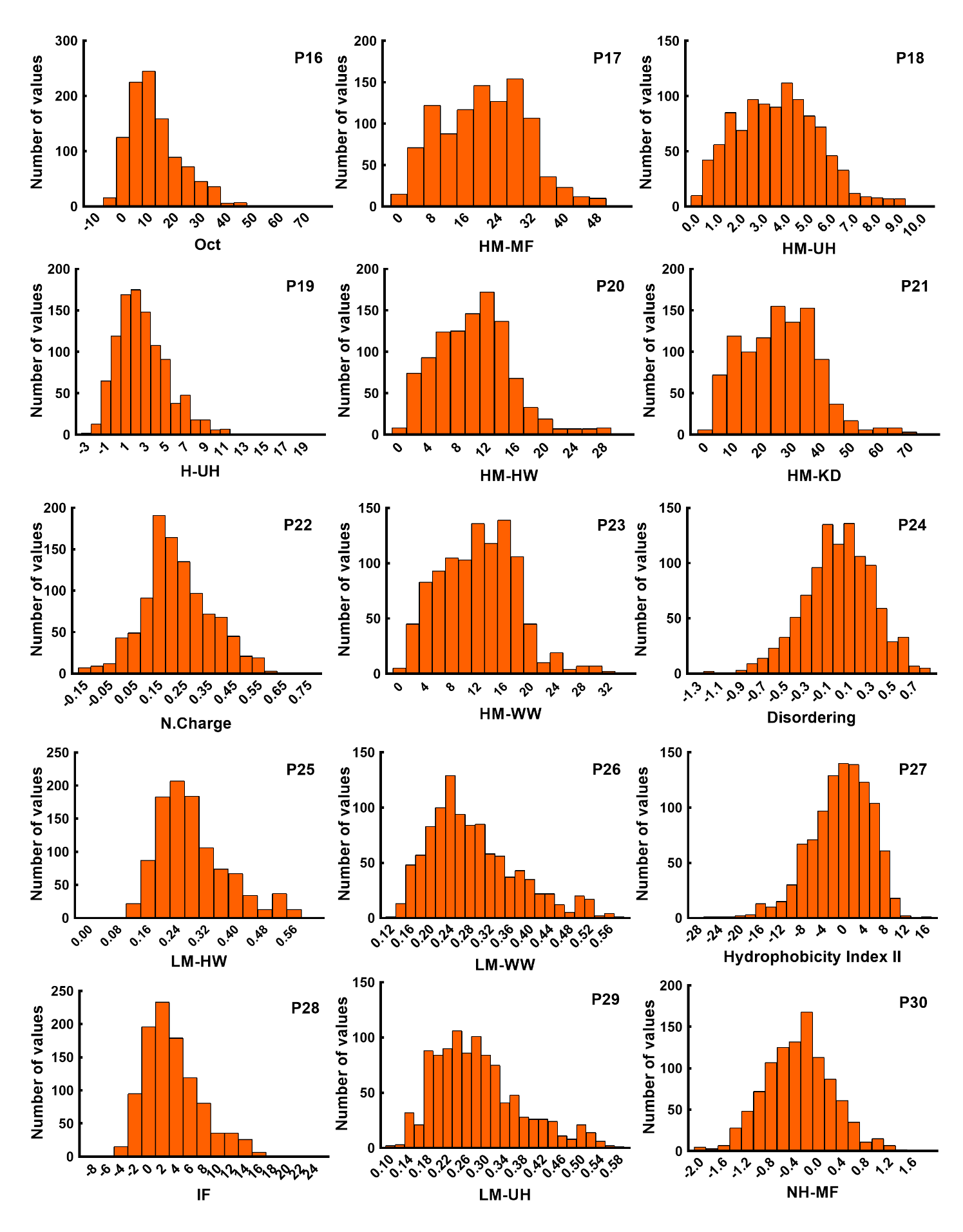


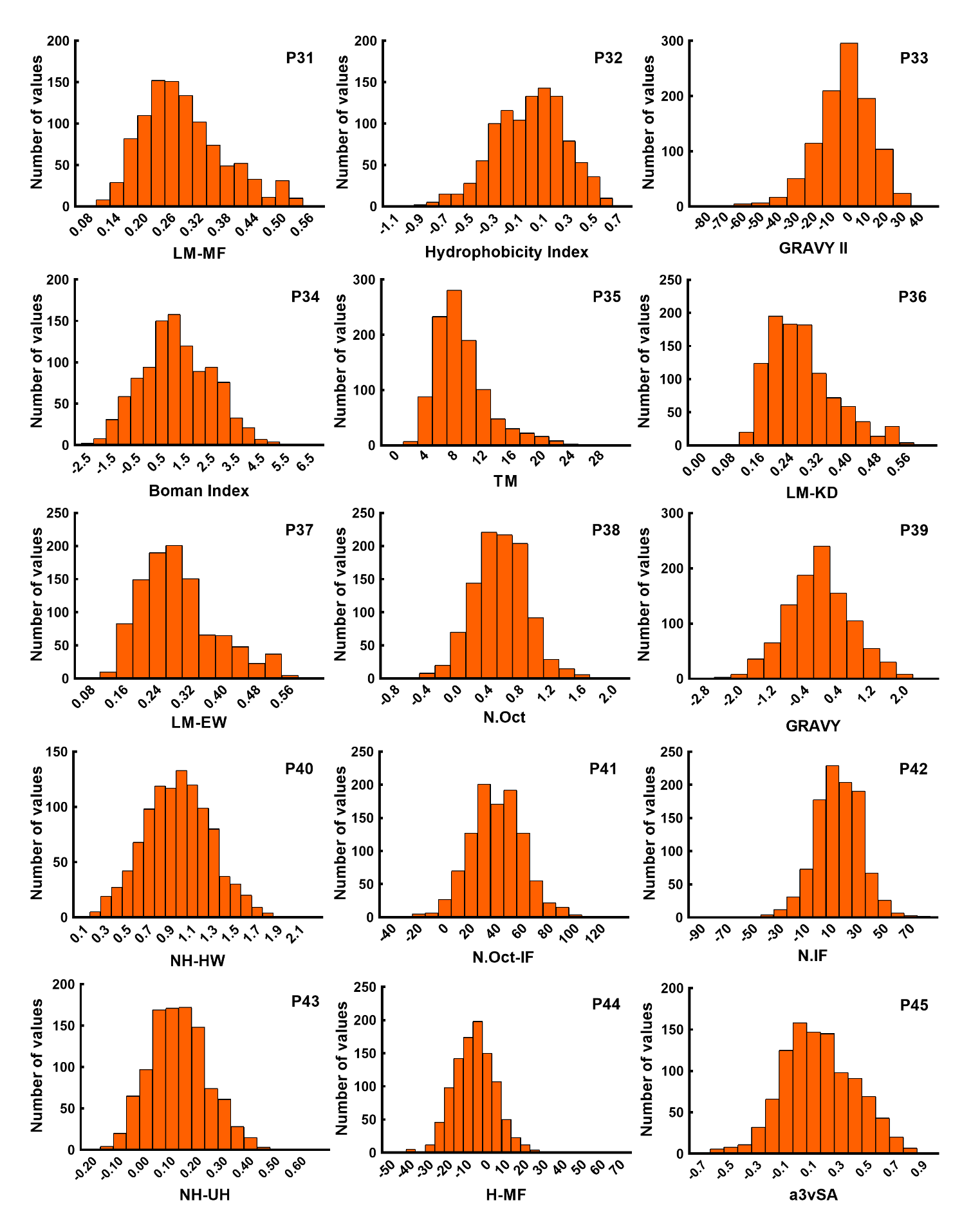


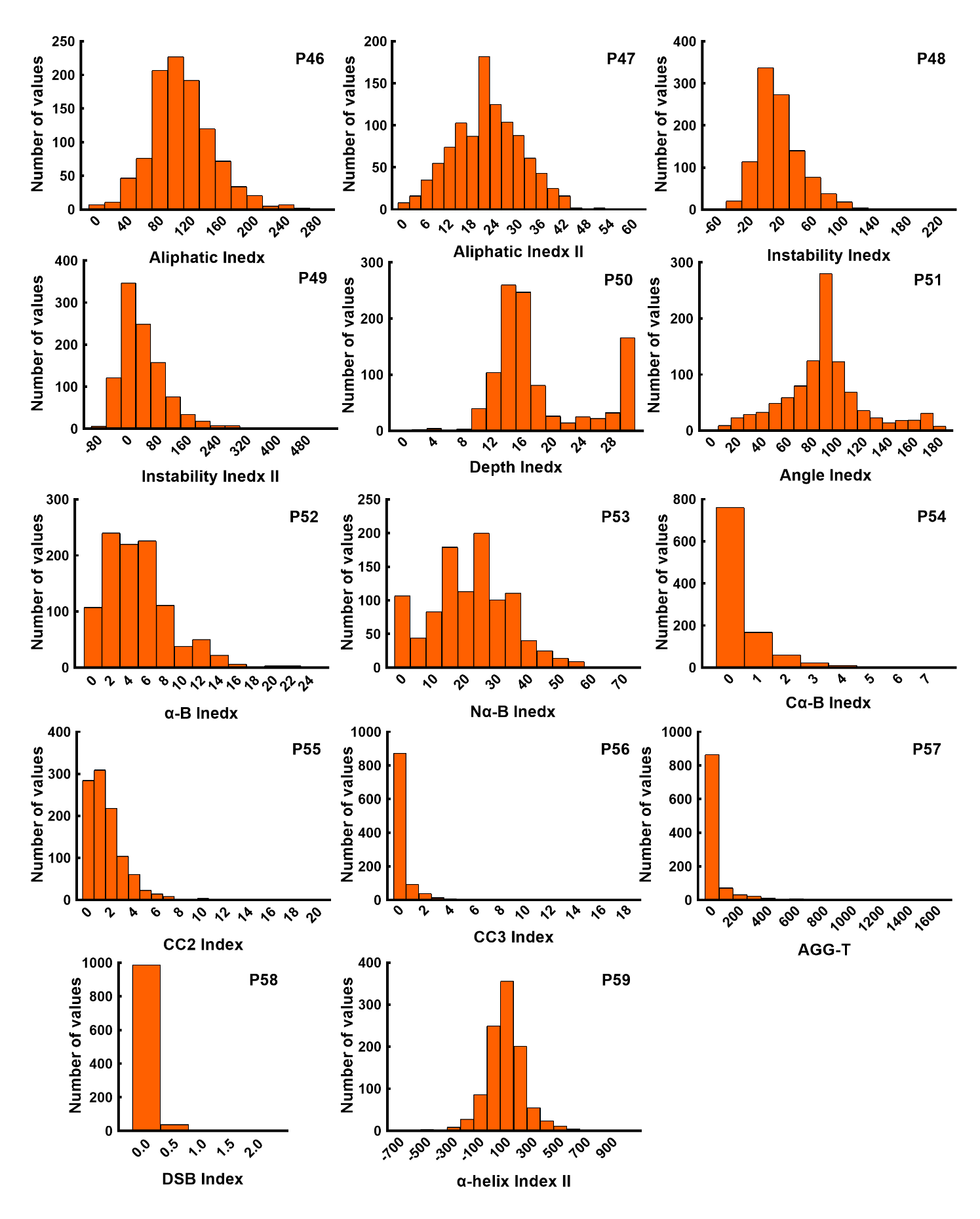


**Supplementary Figure S2**  The histogram distributions of 59 parameters of 1028 α-helix AMPs from DBAASP database.


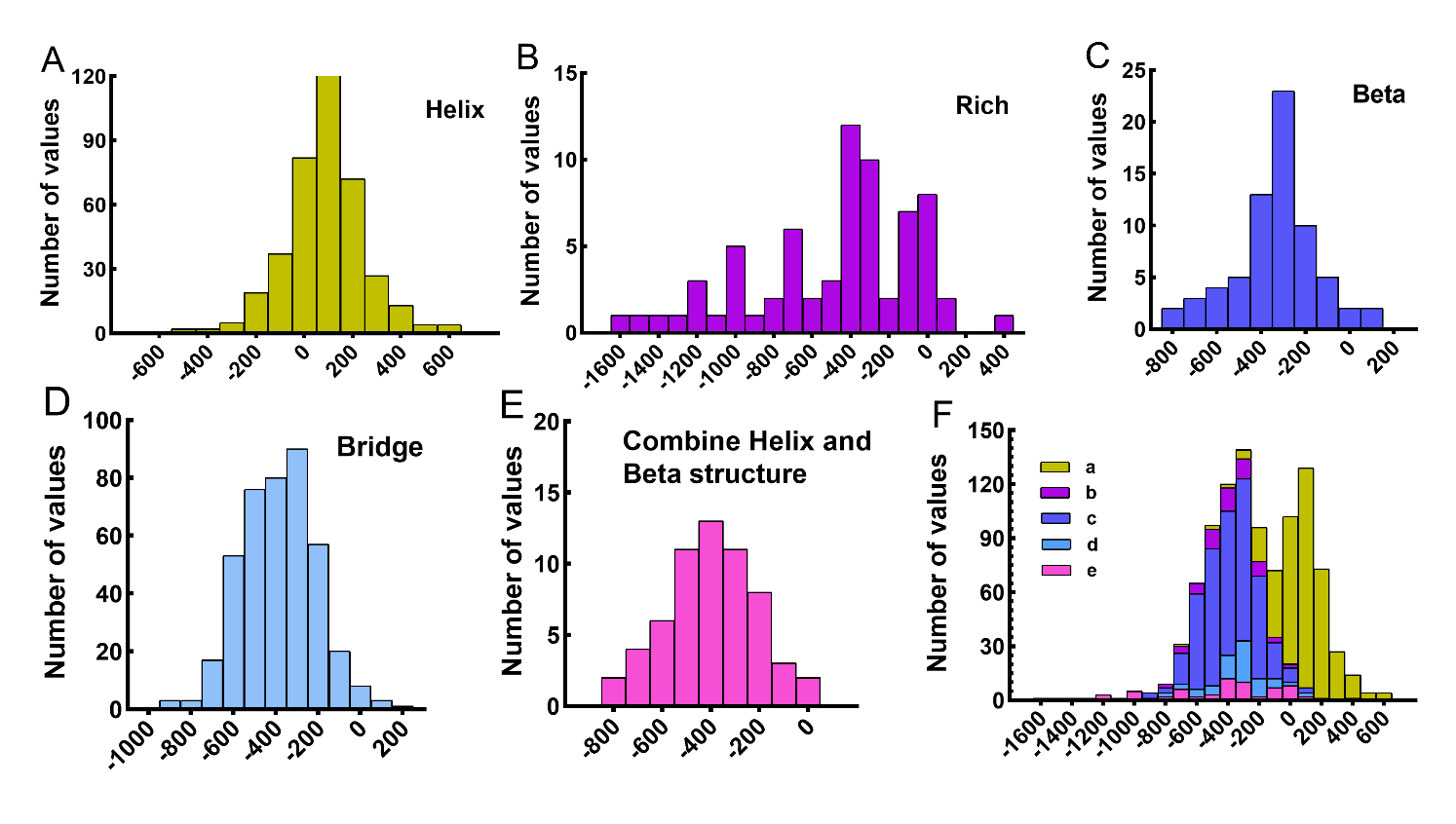


**Supplementary Figure S3**  Histogram distributions of the α-helix Index II (P59) of 999 AMPs. (A) α-helix structure; (B) Rich structure; (C) Beta structure; (D) Bridge structure; (E) Combine Helix and Beta structure; (F) Synthesis of five structures.

## Supplementary Tables

**Supplementary Table S1** The division of 16 datasets of α-helical AMPs in the DBAASP database.

| Name | Abbreviation | Antagonistic object and activity (μM) | Data volume |
| --- | --- | --- | --- |
| Dataset 1 | A1 | *Escherichia coli*, *Pseudomonas aeruginosa* and *Staphylococcus aureus*, all MIC values | 3273 |
| Dataset 2 | A2 | *Escherichia coli*, *Pseudomonas aeruginosa* and *Staphylococcus aureus*, MIC values ≤ 500 | 3088 |
| Dataset 3 | A3 | *Escherichia coli*, *Pseudomonas aeruginosa* and *Staphylococcus aureus*, MIC values ≤ 200 | 3045 |
| Dataset 4 | A4 | *Escherichia coli*, *Pseudomonas aeruginosa* and *Staphylococcus aureus*, MIC values ≤ 100 | 2942 |
| Dataset 5 | E1 | *Escherichia coli*, all MIC values | 1330 |
| Dataset 6 | E2 | *Escherichia coli*, MIC values ≤ 500 | 1293 |
| Dataset 7 | E3 | *Escherichia coli*, MIC values ≤ 200 | 1280 |
| Dataset 8 | E4 | *Escherichia coli*, MIC values ≤ 100 | 1225 |
| Dataset 9 | P1 | *Pseudomonas aeruginosa*, all MIC values | 766 |
| Dataset 10 | P2 | *Pseudomonas aeruginosa*, MIC values ≤ 500 | 760 |
| Dataset 11 | P3 | *Pseudomonas aeruginosa*, MIC values ≤ 200 | 750 |
| Dataset 12 | P4 | *Pseudomonas aeruginosa*, MIC values ≤ 100 | 732 |
| Dataset 13 | S1 | *Staphylococcus aureus*, all MIC values | 1177 |
| Dataset 14 | S2 | *Staphylococcus aureus*, MIC values ≤ 500 | 1035 |
| Dataset 15 | S3 | *Staphylococcus aureus*, MIC values ≤ 200 | 1015 |
| Dataset 16 | S4 | *Staphylococcus aureus*, MIC values ≤ 100 | 985 |

The 3273 data (MIC values) were collected against *E. coli* (1330 data), *P. aeruginosa* (766 data) and *S. aureus* (1177 data). All the 3273 data were divided into 16 datasets according to different MIC groups (all the MIC, MIC ≤ 500 μM, MIC ≤ 200 μM, MIC ≤ 100 μM) and different pathogenic bacteria (all the three pathogenic bacteria, *E. coli*, *P. aeruginosa*, *S. aureus*).

**Supplementary Table S2** The definition and calculation of 59 parameters.

| Name | Abbreviation | Description | References |
| --- | --- | --- | --- |
| Parameter 1 | Net Charge | Net Charge (Charge), was the overall charge of the AMP and was calculated according to the equation proposed by Moore at pH 7.0. | (Moore, 1985) |
| Parameter 2 | pI | Isoelectric Point (pI), was calculated by the software compiled by Lukasz P. Kozlowski (http://isoelectric.ovh.org). | (Kozlowski, 2016) |
| Parameter 3 | Length | Length, was the number of amino acid residues contained in the AMP. | — |
| Parameter 4 | NLM-MF | Normalized Linear Hydrophobic Moment used Moon and Fleming scale (NLM-MF), was the normalized form of LM-MF which was divided by the number of residues of the AMP. | (Vishnepolsky and Pirtskhalava, 2014; Pirtskhalava et al., 2016) |
| Parameter 5 | α-helix | α-Helix Index (α-helix), was evaluated the overall level of conformational parameter for the helix by summing over all values of conformational parameter for the helix (Pa). | (Chou and Fasman, 1974a; Chou and Fasman, 1974b) |
| Parameter 6 | H-HW | Hydrophobicity used Hessa and White scale (H-HW), was the denormalized form of NH-HW. | (Vishnepolsky and Pirtskhalava, 2014; Pirtskhalava et al., 2016) |
| Parameter 7 | NLM-HW | Normalized Linear Hydrophobic Moment used Hessa and White scale (NLM-HW), was the normalized form of LM-HW which was divided by the number of residues of the AMP. | (Vishnepolsky and Pirtskhalava, 2014; Pirtskhalava et al., 2016) |
| Parameter 8 | NLM-WW | Normalized Linear Hydrophobic Moment used Wimley and White scale (NLM-WW), was the normalized form of LM-WW which was divided by the number of residues of the AMP. | (Vishnepolsky and Pirtskhalava, 2014; Pirtskhalava et al., 2016) |
| Parameter 9 | NLM-UH | Normalized Linear Hydrophobic Moment used Unified Hydrophobicity scale (NLM-UH), was the normalized form of LM-UH which was divided by the number of residues of the AMP. | (Vishnepolsky and Pirtskhalava, 2014; Pirtskhalava et al., 2016) |
| Parameter 10 | HM | Hydrophobic Moment (HM), was used Eisenberg and Weiss scale and calculated according to the calculation method proposed by Eisenberg et al. | (Eisenberg et al., 1982) |
| Parameter 11 | NLM-EW | Normalized Linear Hydrophobic Moment used Eisenberg and Weiss scale (NLM-EW), was the normalized form of LM-EW which was divided by the number of residues of the AMP. | (Vishnepolsky and Pirtskhalava, 2014; Pirtskhalava et al., 2016) |
| Parameter 12 | NLM-KD | Normalized Linear Hydrophobic Moment used Kyte and Doolittle scale (NLM-KD), was the normalized form of LM-KD which was divided by the number of residues of the AMP. | (Vishnepolsky and Pirtskhalava, 2014; Pirtskhalava et al., 2016) |
| Parameter 13 | HM-EW | Hydrophobic Moment used Eisenberg and Weiss scale (HM-EW), was optimized by Boris Vishnepolsky and Malak Pirtskhalava and calculated applying DBAASP website. | (Eisenberg et al., 1984b; Vishnepolsky and Pirtskhalava, 2014; Pirtskhalava et al., 2016) |
| Parameter 14 | Oct-IF | Oct-IF Index (Oct-IF), was free energy differences between the octanol and IF scales, using for identifying segments that tend to prefer a transbilayer helix conformation, and was calculated by the software Membrane Protein Explorer (MPEx). | (Snider et al., 2009) |
| Parameter 15 | Boman II | Boman Index II (Boman II), was the unnormalized Boman Index and calculated according to the method proposed by H. G. Boman, which was summed over all free energies values of the respective side chains used the Radzeka and Wolfenden scale. | (Radzicka and Wolfenden, 1988; Boman, 2003) |
| Parameter 16 | Oct | Octanol Index (Oct), representing the hydrophily of the residues’ free energy of transfer from water to the bilayer hydrocarbon core, was calculated by the software Membrane Protein Explorer (MPEx). | (Snider et al., 2009) |
| Parameter 17 | HM-MF | Hydrophobic Moment used Moon and Fleming scale (HM-MF), was optimized by Boris Vishnepolsky and Malak Pirtskhalava, and calculated applying DBAASP website. | (Moon and Fleming, 2011; Vishnepolsky and Pirtskhalava, 2014; Pirtskhalava et al., 2016) |
| Parameter 18 | HM-UH | Hydrophobic Moment used Unified Hydrophobicity scale (HM-UH), was optimized by Boris Vishnepolsky and Malak Pirtskhalava, and calculated applying DBAASP website. | (Koehler et al., 2009; Vishnepolsky and Pirtskhalava, 2014; Pirtskhalava et al., 2016) |
| Parameter 19 | H-UH | Hydrophobicity used Unified Hydrophobicity scale (H-UH), was the denormalized form of NH-UH. | (Vishnepolsky and Pirtskhalava, 2014; Pirtskhalava et al., 2016) |
| Parameter 20 | HM-HW | Hydrophobic Moment used Hessa and White scale (HM-HW), was optimized by Boris Vishnepolsky and Malak Pirtskhalava, and calculated applying DBAASP website. | (Hessa et al., 2005; Vishnepolsky and Pirtskhalava, 2014; Pirtskhalava et al., 2016) |
| Parameter 21 | HM-KD | Hydrophobic Moment used Kyte and Doolittle scale (HM-KD), was optimized by Boris Vishnepolsky and Malak Pirtskhalava, and calculated applying DBAASP website. | (Kyte and Doolittle, 1982; Vishnepolsky and Pirtskhalava, 2014; Pirtskhalava et al., 2016) |
| Parameter 22 | N.Charge | Normalized Charge (N.charge), was the full net charge divided by the total number of residues of the AMP. | — |
| Parameter 23 | HM-WW | Hydrophobic Moment used Wimley and White scale (HM-WW), was optimized by Boris Vishnepolsky and Malak Pirtskhalava, and calculated applying DBAASP website. | (Wimley and White, 1996; Vishnepolsky and Pirtskhalava, 2014; Pirtskhalava et al., 2016) |
| Parameter 24 | Disordering | Disordering Index (Disordering), representing the degree of disordering of short peptides in water environment according to the Uversky’s rule and was calculated applying DBAASP website. | (Uversky et al., 2000; Vishnepolsky and Pirtskhalava, 2014; Pirtskhalava et al., 2016) |
| Parameter 25 | LM-HW | Linear Moment used Hessa and White scale (LM-HW), was proposed by Boris Vishnepolsky and Malak Pirtskhalava, and calculated applying DBAASP website. | (Vishnepolsky and Pirtskhalava, 2014; Pirtskhalava et al., 2016) |
| Parameter 26 | LM-WW | Linear Moment used Wimley and White scale (LM-WW), was proposed by Boris Vishnepolsky and Malak Pirtskhalava, and calculated applying DBAASP website. | (Vishnepolsky and Pirtskhalava, 2014; Pirtskhalava et al., 2016) |
| Parameter 27 | H II | Hydrophobicity Index II (H II), was the unnormalized Hydrophobicity Index using Eisenberg and Weiss scale, was not normalized against the length of the AMP. | (Eisenberg et al., 1984a) |
| Parameter 28 | IF | Interfacial HydrophobicityIndex (IF), representing the hydrophobicity by measuring the residues’ free energy of transfer from water to a phosphocholine bilayer interface, was calculated by the software Membrane Protein Explorer (MPEx). | (Snider et al., 2009) |
| Parameter 29 | LM-UH | Linear Moment used Unified Hydrophobicity scale (LM-UH), was proposed by Boris Vishnepolsky and Malak Pirtskhalava, and calculated applying DBAASP website. | (Vishnepolsky and Pirtskhalava, 2014; Pirtskhalava et al., 2016) |
| Parameter 30 | NH-MF | Normalized Hydrophobicity used Moon and Fleming scale (NH-MF), was optimized by Boris Vishnepolsky and Malak Pirtskhalava and calculated applying DBAASP website. | (Moon and Fleming, 2011; Vishnepolsky and Pirtskhalava, 2014; Pirtskhalava et al., 2016) |
| Parameter 31 | LM-MF | Linear Moment used Moon and Fleming scale (LM-MF), was proposed by Boris Vishnepolsky and Malak Pirtskhalava, and calculated applying DBAASP website. | (Pirtskhalava et al., 2016) |
| Parameter 32 | H | Hydrophobicity Index (H), was calculated according to the method proposed by Eisenberg and Weiss . | (Eisenberg et al., 1984a) |
| Parameter 33 | GRAVY II | GRAVY II, was the unnormalized form of GRAVY, was not normalized against the length of peptide sequence. | (Kyte and Doolittle, 1982) |
| Parameter 34 | Boman | Protein-Binding Potential Index (Boman), was calculated according to the method proposed by H. G. Boman. | (Radzicka and Wolfenden, 1988; Boman, 2003) |
| Parameter 35 | TM | Translocon TM Index (TM), was analyzed the hydropathy of transmembrane proteins based on transmembrane-helix recognition using the software Membrane Protein Explorer (MPEx) (https://en.bio-soft.net/protein/MPEx.html). | (Snider et al., 2009) |
| Parameter 36 | LM-KD | Linear Moment used Kyte and Doolittle scale (LM-KD), was proposed by Boris Vishnepolsky and Malak Pirtskhalava, and calculated applying DBAASP website. | (Vishnepolsky and Pirtskhalava, 2014; Pirtskhalava et al., 2016) |
| Parameter 37 | LM-EW | Linear Moment used Eisenberg and Weiss scale (LM-EW), was proposed by Boris Vishnepolsky and Malak Pirtskhalava, and calculated applying DBAASP website. | (Vishnepolsky and Pirtskhalava, 2014; Pirtskhalava et al., 2016) |
| Parameter 38 | N.Oct | Normalized Octanol Index (N.Oct), was the normalized Octanol Index against the length of the AMP. | (Snider et al., 2009) |
| Parameter 39 | GRAVY | Grand Average of Hydrophily (GRAVY), was calculated as the sum of Hydrophilic values of all amino acids of peptide sequence according to the Kyte and Doolittle scale, and divided by the length of the sequence. | (Kyte and Doolittle, 1982) |
| Parameter 40 | NH-HW | Normalized Hydrophobicity used Hessa and White scale (NH-HW), was optimized by Boris Vishnepolsky and Malak Pirtskhalava and calculated applying DBAASP website. | (Hessa et al., 2005; Vishnepolsky and Pirtskhalava, 2014; Pirtskhalava et al., 2016) |
| Parameter 41 | N.Oct-IF | Normalized Oct-IF Index (N.Oct-IF), was the normalized Oct-IF Index against the length of the AMP. | — |
| Parameter 42 | N.IF | Normalized Interfacial Hydrophobicity (N.IF) Index, was the normalized IF Index against the length of AMP. | — |
| Parameter 43 | NH-UH | Normalized Hydrophobicity used Unified Hydrophobicity scale (NH-UH), was optimized by Boris Vishnepolsky and Malak Pirtskhalava and calculated applying DBAASP website. | (Koehler et al., 2009; Vishnepolsky and Pirtskhalava, 2014; Pirtskhalava et al., 2016) |
| Parameter 44 | H-MF | Hydrophobicity used Moon and Fleming scale (H-MF), was the denormalized form of NH-MF. | (Vishnepolsky and Pirtskhalava, 2014; Pirtskhalava et al., 2016) |
| Parameter 45 | a3vSA | Amino acid aggregation propensity value Sequence Average (a3vSA), was the normalized a3v against the length of AMP. | (de Groot et al., 2012) |
| Parameter 46 | A | Aliphatic Index (A), was evaluated the thermal stability of proteins and calculated according to the method proposed by Ikai. | (Ikai, 1980) |
| Parameter 47 | A II | Aliphatic Index II (A II), was the unnormalized Aliphatic Index and calculated by summing over the relative volumes of aliphatic side chains of alanine, valine, isoleucine, and leucine according to the following formula: Aliphatic Index II = N_A_ + 2.9N_V_ + 3.9(N_I_ + N_L_), where N_A_, N_V_, N_I_ and N_L_ were the number of alanine, valine, isoleucine and leucine contained in the peptide, respectively. | (Ikai, 1980) |
| Parameter 48 | Instability | Instability Index (Instability), was the unnormalized Instability Index II and calculated by summing over all dipeptide instability weight value. | (Guruprasad et al., 1990) |
| Parameter 49 | Instability II | Instability Index II (Instability II), was evaluated the metabolic stability of proteins with features of their primary sequence and calculated according to the method proposed by Guruprasad et al.. | (Guruprasad et al., 1990) |
| Parameter 50 | Depth | Penetration Depth Index (Depth), was evaluated the penetration depth of peptide binding to lipid bilayer and calculated applying DBAASP website. | (Senes et al., 2007; Vishnepolsky and Pirtskhalava, 2014; Pirtskhalava et al., 2016) |
| Parameter 51 | Angle | Penetration Angle Index (Angle), was evaluated the penetration angle of peptide binding to lipid bilayer and calculated applying DBAASP website. | (Senes et al., 2007; Vishnepolsky and Pirtskhalava, 2014; Pirtskhalava et al., 2016) |
| Parameter 52 | α-B | α-B Index (α-B), was α-helix broker index. Asparagine (N), tyrosine (Y), proline (P) and glycine (G) are usual α-breakers as hindrance when α-helix formation. The α-helix broker Index was used to evaluate the hindering effect of α-breakers, and calculated by summing over all assigned value of α-breakers in the peptide sequence which was used as a measurement of the resistance to the formation of α-helix structure. The assigned values of α-breaker was expressed as V*_αb_* = (1-*P_a_*)/(1-*P_a_*_(N)_), in which, *P_a_* was the conformational parameter value for the helix. So N, Y, P and G were assigned values of 1, 1.44, 1.52 and 1.74 separately based on the previous formula. | (Chou and Fasman, 1974a; Chou and Fasman, 1974b) |
| Parameter 53 | N.α-B | Nα-B Index (Nα-B), the normalized α-helix broker Index, was α helix broker index divided by the total number of residues of the AMP and then multiplied by 100. | — |
| Parameter 54 | Cα-B | Cα-B Index (Cα-B), consecutive α-helix breakers index, representing the resistance to form α-helix structure. Four amino acids, asparagine (N), tyrosine (Y), proline (P) and glycine (G) are usual as α-breakers, which can form 16 patterns for two consecutive α-breakers amino acid residues, i.e., GG, PG, YG, NG, GP, PP, YP, NP, GY, PY, YY, NY, GN, PN, YN and NN. Each pattern was assigned value of 1. Cα-B Index was calculated by summing over all assigned value of the consecutive α-breakers in the AMP. | — |
| Parameter 55 | CC2 | CC2 Index (CC2), index of two consecutive amino acid residues with identical charges. The α-helix formation is usually hindered to a certain extent under the existence of the consecutive amino acid residues with the same type of charges. Five charged amino acids comprise positively charged amino acids, arginine (R), lysine (K), histidine (H). So do the negatively charged amino acids, aspartic acid (D), glutamic acid (E). Totally 13 patterns of two consecutive amino acid residues with identical charges were assigned value of 1, i.e., RR, RK, RH, KR, KK, KH, HR, HK, HH, DD, DE, ED and EE. The CC2 index was computed by summing these values of dipeptides in the peptide sequence, and the result was used as a measurement of the resistance to the formation of α-helix structure. | — |
| Parameter 56 | CC3 | CC3 Index (CC3), index of three consecutive amino acid residues with identical charges. Similar to the CC2 Index, the CC3 Index was used to evaluate the hindering formation α-helix effect when three consecutive amino acid residues with identical charges. Arginine (R), lysine (K) and histidine (H) comprise positively charges, while aspartic acid (D) and glutamic acid (E) comprise negatively charges. Totally 33 patterns of three consecutive amino acid residues with the same type of charges were assigned values of 1, i.e., RRR, RRK, RRH, RKR, RKK, RKH, RHR, RHK, RHH, KRR, KRK, KRH, KKR, KKK, KKH, KHR, KHK, KHH, HRR, HRK, HRH, HKR, HKK, HKH, HHR, HHK, HHH, DDD, DDE, DEE, EEE, EED, EDD. The CC3 index was computed by summing these values of tripeptide, and the result was used as a measurement of the resistance to the formation of α-helix structure. | — |
| Parameter 57 | AGG-T | Aggregation Index of TANGO (AGG-T), was calculated intrinsic aggregation propensity of polypeptides by employing the TANGO software (http://tango.crg.es/). | (Conchillo-Solé et al., 2007) |
| Parameter 58 | DSB | Disulfide Bond index (DSB), was defined as the sum of all over of cysteine residue value. and used to evaluate potential disulfide bond. Here, every cysteine residue was assigned value 0.5. | — |
| Parameter 59 | α-helix II | α-helix Index II (α-helix II), was used to evaluate the α-helical conformation distinguish from other conformations by summing over all the assigned values of 20 common amino acids (H’) evolved from conformational parameter for the helix (*P_a_*) by according to the formula H’ = 100*(*P_a_* -1). | — |

**Supplementary Table S3** The correlation coefficients of 58 parameters to MIC values.

| Parameter | Name | A1 | A2 | A3 | A4 | E1 | E2 | E3 | E4 | P1 | P2 | P3 | P4 | S1 | S2 | S3 | S4 | AVCC |
| --- | --- | --- | --- | --- | --- | --- | --- | --- | --- | --- | --- | --- | --- | --- | --- | --- | --- | --- |
| P1 | Net Charge | -0.5332^a^ | -0.4471^a^ | -0.4381^a^ | -0.4102^a^ | -0.5585^a^ | -0.5219^a^ | -0.514^a^ | -0.4854^a^ | -0.4892^a^ | -0.4772^a^ | -0.4683^a^ | -0.4448^a^ | -0.5202^a^ | -0.3025^a^ | -0.2897^a^ | -0.2624^a^ | -0.447 |
| P2 | Pi | -0.4393^a^ | -0.3368^a^ | -0.326^a^ | -0.3121^a^ | -0.4102^a^ | -0.361^a^ | -0.3576^a^ | -0.333^a^ | -0.3193^a^ | -0.3036^a^ | -0.2852^a^ | -0.2741^a^ | -0.5331^a^ | -0.3272^a^ | -0.3115^a^ | -0.3108^a^ | -0.346 |
| P3 | Length | -0.4255^a^ | -0.3224^a^ | -0.3125^a^ | -0.2921^a^ | -0.4089^a^ | -0.3621^a^ | -0.3544^a^ | -0.3481^a^ | -0.3906^a^ | -0.3773^a^ | -0.378^a^ | -0.349^a^ | -0.4477^a^ | -0.2038^a^ | -0.1809^a^ | -0.1486^a^ | -0.331 |
| P4 | NLM-MF | 0.4282^a^ | 0.3273^a^ | 0.3148^a^ | 0.2852^a^ | 0.4508^a^ | 0.4069^a^ | 0.3962^a^ | 0.38^a^ | 0.3879^a^ | 0.3742^a^ | 0.3742^a^ | 0.3456^a^ | 0.4059^a^ | 0.1497^a^ | 0.1217^a^ | 0.0772^c^ | 0.327 |
| P5 | α-helix | -0.4136^a^ | -0.3115^a^ | -0.301^a^ | -0.28^a^ | -0.399^a^ | -0.3527^a^ | -0.3441^a^ | -0.3383^a^ | -0.382^a^ | -0.3686^a^ | -0.3686^a^ | -0.3388^a^ | -0.4307^a^ | -0.1877^a^ | -0.1649^a^ | -0.1306^a^ | -0.32 |
| P6 | H-HW | -0.3979^a^ | -0.3173^a^ | -0.309^a^ | -0.2816^a^ | -0.4187^a^ | -0.3841^a^ | -0.3792^a^ | -0.3625^a^ | -0.3573^a^ | -0.3446^a^ | -0.3398^a^ | -0.3085^a^ | -0.3626^a^ | -0.1812^a^ | -0.1638^a^ | -0.1257^a^ | -0.315 |
| P7 | NLM-HW | 0.4087^a^ | 0.3074^a^ | 0.2938^a^ | 0.2689^a^ | 0.4047^a^ | 0.359^a^ | 0.3474^a^ | 0.3377^a^ | 0.4012^a^ | 0.3887^a^ | 0.3882^a^ | 0.3585^a^ | 0.4017^a^ | 0.1515^a^ | 0.122^a^ | 0.0839^b^ | 0.314 |
| P8 | NLM-WW | 0.4069^a^ | 0.3034^a^ | 0.2909^a^ | 0.2663^a^ | 0.4067^a^ | 0.3426^a^ | 0.3493^a^ | 0.3426^a^ | 0.4198^a^ | 0.4073^a^ | 0.4074^a^ | 0.3799^a^ | 0.385^a^ | 0.1215^a^ | 0.0933^b^ | 0.0478^n^ | 0.308 |
| P9 | NLM-UH | 0.3983^a^ | 0.2942^a^ | 0.2796^a^ | 0.2553^a^ | 0.4095^a^ | 0.3638^a^ | 0.3517^a^ | 0.3455^a^ | 0.3696^a^ | 0.3558^a^ | 0.3534^a^ | 0.3218^a^ | 0.3843^a^ | 0.1256^a^ | 0.095^b^ | 0.0548^n^ | 0.294 |
| P10 | HM | -0.3961^a^ | -0.2914^a^ | -0.2792^a^ | -0.2416^a^ | -0.4311^a^ | -0.3875^a^ | -0.3763^a^ | -0.3458^a^ | -0.3194^a^ | -0.3058^a^ | -0.3005^a^ | -0.2641^a^ | -0.3967^a^ | -0.137^a^ | -0.1138^a^ | -0.071^c^ | -0.291 |
| P11 | NLM-EW | 0.3905^a^ | 0.2869^a^ | 0.2734^a^ | 0.2465^a^ | 0.4042^a^ | 0.36^a^ | 0.3491^a^ | 0.3412^a^ | 0.3658^a^ | 0.3516^a^ | 0.3493^a^ | 0.3165^a^ | 0.3667^a^ | 0.1048^a^ | 0.0747^c^ | 0.0305^n^ | 0.286 |
| P12 | NLM-KD | 0.3855^a^ | 0.2794^a^ | 0.2647^a^ | 0.237^a^ | 0.3923^a^ | 0.3458^a^ | 0.3342^a^ | 0.3236^a^ | 0.3405^a^ | 0.3264^a^ | 0.3236^a^ | 0.2902^a^ | 0.3842^a^ | 0.1243^a^ | 0.093^b^ | 0.0507^n^ | 0.278 |
| P13 | HM-EW | -0.3732^a^ | -0.2759^a^ | -0.2577^a^ | -0.2316^a^ | -0.3874^a^ | -0.3466^a^ | -0.3339^a^ | -0.3166^a^ | -0.3713^a^ | -0.3595^a^ | -0.3454^a^ | -0.3135^a^ | -0.3372^a^ | -0.0828^b^ | -0.0485^n^ | -0.0186^n^ | -0.271 |
| P14 | Oct-IF | -0.3626^a^ | -0.2741^a^ | -0.2659^a^ | -0.2376^a^ | -0.3965^a^ | -0.3575^a^ | -0.3483^a^ | -0.3314^a^ | -0.3245^a^ | -0.3142^a^ | -0.3128^a^ | -0.2832^a^ | -0.3126^a^ | -0.1043^a^ | -0.0902^b^ | -0.0489^n^ | -0.27 |
| P15 | Boman II | 0.293^a^ | 0.2716^a^ | 0.2661^a^ | 0.2436^a^ | 0.3426^a^ | 0.3321^a^ | 0.331^a^ | 0.3063^a^ | 0.2287^a^ | 0.2194^a^ | 0.2113^a^ | 0.1927^a^ | 0.2504^a^ | 0.2162^a^ | 0.2068^a^ | 0.1848^a^ | 0.256 |
| P16 | Oct | -0.3249^a^ | -0.2668^a^ | -0.2574^a^ | -0.2287^a^ | -0.3723^a^ | -0.3472^a^ | -0.3365^a^ | -0.3208^a^ | -0.3295^a^ | -0.3209^a^ | -0.3199^a^ | -0.2873^a^ | -0.2221^a^ | -0.0898^b^ | -0.0739^c^ | -0.032^n^ | -0.256 |
| P17 | HM-MF | -0.3598^a^ | -0.2552^a^ | -0.2368^a^ | -0.2115^a^ | -0.3638^a^ | -0.3183^a^ | -0.3055^a^ | -0.2906^a^ | -0.3481^a^ | -0.3366^a^ | -0.32^a^ | -0.2867^a^ | -0.34^a^ | -0.0761^c^ | -0.0442^n^ | -0.0146^n^ | -0.253 |
| P18 | HM-UH | -0.3541^a^ | -0.2517^a^ | -0.2326^a^ | -0.2082^a^ | -0.3707^a^ | -0.327^a^ | -0.3119^a^ | -0.2993^a^ | -0.3652^a^ | -0.3535^a^ | -0.3393^a^ | -0.307^a^ | -0.3081^a^ | -0.035^n^ | 0.0004^n^ | 0.0326^n^ | -0.252 |
| P19 | H-UH | -0.2845^a^ | -0.2582^a^ | -0.2529^a^ | -0.2289^a^ | -0.3636^a^ | -0.3519^a^ | -0.3478^a^ | -0.328^a^ | -0.2892^a^ | -0.2818^a^ | -0.2754^a^ | -0.2529^a^ | -0.141^a^ | -0.0925^b^ | -0.0858^b^ | -0.0579^n^ | -0.24 |
| P20 | HM-HW | -0.3379^a^ | -0.2381^a^ | -0.2197^a^ | -0.1969^a^ | -0.3416^a^ | -0.2989^a^ | -0.2839^a^ | -0.2704^a^ | -0.3544^a^ | -0.3431^a^ | -0.33^a^ | -0.3018^a^ | -0.3041^a^ | -0.0447^n^ | -0.0126^n^ | 0.0156^n^ | -0.239 |
| P21 | HM-KD | -0.328^a^ | -0.226^a^ | -0.2072^a^ | -0.1857^a^ | -0.3188^a^ | -0.2745^a^ | -0.2599^a^ | -0.2479^a^ | -0.341^a^ | -0.3293^a^ | -0.3151^a^ | -0.2862^a^ | -0.3142^a^ | -0.0578^n^ | -0.0254^n^ | 0.0003^n^ | -0.227 |
| P22 | N.Charge | -0.3336^a^ | -0.2152^a^ | -0.2047^a^ | -0.1982^a^ | -0.3092^a^ | -0.254^a^ | -0.2444^a^ | -0.2256^a^ | -0.1987^a^ | -0.185^a^ | -0.1639^a^ | -0.1679^a^ | -0.4219^a^ | -0.1684^a^ | -0.1625^a^ | -0.1661^a^ | -0.226 |
| P23 | HM-WW | -0.3124^a^ | -0.2147^a^ | -0.1979^a^ | -0.1781^a^ | -0.3027^a^ | -0.2586^a^ | -0.2447^a^ | -0.2383^a^ | -0.3409^a^ | -0.3304^a^ | -0.3185^a^ | -0.2913^a^ | -0.2855^a^ | -0.0444^n^ | -0.0174^n^ | 0.0126^n^ | -0.22 |
| P24 | Disordering | 0.2596^a^ | 0.2254^a^ | 0.2192^a^ | 0.193^a^ | 0.3039^a^ | 0.2898^a^ | 0.2882^a^ | 0.2525^a^ | 0.1859^a^ | 0.1735^a^ | 0.1555^a^ | 0.1365^a^ | 0.2145^a^ | 0.151^a^ | 0.1457^a^ | 0.1284^a^ | 0.208 |
| P25 | LM-HW | 0.2969^a^ | 0.2053^a^ | 0.1903^a^ | 0.1631^a^ | 0.2891^a^ | 0.2473^a^ | 0.2338^a^ | 0.2214^a^ | 0.2827^a^ | 0.2721^a^ | 0.2652^a^ | 0.2283^a^ | 0.2904^a^ | 0.0798^c^ | 0.0537^n^ | 0.0177^n^ | 0.204 |
| P26 | LM-WW | 0.2867^a^ | 0.1948^a^ | 0.1802^a^ | 0.1536^a^ | 0.2937^a^ | 0.2494^a^ | 0.2352^a^ | 0.2241^a^ | 0.3134^a^ | 0.3023^a^ | 0.2964^a^ | 0.2673^a^ | 0.2336^a^ | 0.019^n^ | -0.0058^n^ | -0.0508^n^ | 0.202 |
| P27 | H II | 0.1996^a^ | 0.2182^a^ | 0.2158^a^ | 0.1962^a^ | 0.2868^a^ | 0.2951^a^ | 0.2954^a^ | 0.2666^a^ | 0.1861^a^ | 0.1817^a^ | 0.1713^a^ | 0.1623^a^ | 0.0755^b^ | 0.1255^a^ | 0.1254^a^ | 0.1111^a^ | 0.195 |
| P28 | IF | -0.2053^a^ | -0.2223^a^ | -0.2165^a^ | -0.1913^a^ | -0.2857^a^ | -0.2929^a^ | -0.2841^a^ | -0.2726^a^ | -0.2871^a^ | -0.2831^a^ | -0.2852^a^ | -0.2503^a^ | -0.0177^n^ | -0.0591^n^ | -0.0502^n^ | -0.0155^n^ | -0.192 |
| P29 | LM-UH | 0.2785^a^ | 0.1827^a^ | 0.1655^a^ | 0.1408^a^ | 0.2952^a^ | 0.2514^a^ | 0.2354^a^ | 0.2278^a^ | 0.2665^a^ | 0.254^a^ | 0.2433^a^ | 0.2077^a^ | 0.2469^a^ | 0.0193^n^ | -0.0079^n^ | -0.0446^n^ | 0.187 |
| P30 | NH-MF | -0.2325^a^ | -0.2089^a^ | -0.2009^a^ | -0.1664^a^ | -0.3044^a^ | -0.2976^a^ | -0.2879^a^ | -0.2524^a^ | -0.2192^a^ | -0.2129^a^ | -0.2023^a^ | -0.1741^a^ | -0.1211^a^ | -0.0644^c^ | -0.0571^n^ | -0.0223^n^ | -0.184 |
| P31 | LM-MF | 0.2854^a^ | 0.189^a^ | 0.1741^a^ | 0.1411^a^ | 0.3195^a^ | 0.2734^a^ | 0.2593^a^ | 0.2348^a^ | 0.2156^a^ | 0.2005^a^ | 0.1928^a^ | 0.1596^a^ | 0.2594^a^ | 0.046^n^ | 0.0218^n^ | -0.018^n^ | 0.182 |
| P32 | H | 0.1813^a^ | 0.1994^a^ | 0.1955^a^ | 0.1719^a^ | 0.2547^a^ | 0.2628^a^ | 0.2643^a^ | 0.2311^a^ | 0.16^a^ | 0.1499^a^ | 0.1336^a^ | 0.118^b^ | 0.0771^b^ | 0.1279^a^ | 0.1261^a^ | 0.1103^a^ | 0.173 |
| P33 | GRAVY II | 0.1732^a^ | 0.1912^a^ | 0.1896^a^ | 0.1669^a^ | 0.2539^a^ | 0.2631^a^ | 0.2655^a^ | 0.2373^a^ | 0.152^a^ | 0.1438^a^ | 0.1354^a^ | 0.1184^b^ | 0.0574^c^ | 0.1078^a^ | 0.1063^a^ | 0.0877^b^ | 0.166 |
| P34 | Boman | -0.1444^a^ | -0.1951^a^ | -0.1923^a^ | -0.1718^a^ | -0.2177^a^ -0.2177^a^ | -0.2411^a^ | -0.2456^a^ | -0.215^a^ | -0.1385^a^ | -0.1287^a^ | -0.1138^b^ | -0.0995^b^ | -0.0524^n^ | -0.1678 | -0.1646 | -0.153 | -0.162 |
| P35 | TM | -0.1496^a^ | -0.1648^a^ | -0.1646^a^ | -0.1489^a^ | -0.2699^a^ | -0.2781^a^ | -0.2748^a^ | -0.265^a^ | -0.1916^a^ | -0.1898^a^ | -0.1942^a^ | -0.1786^a^ | 0.0687^c^ | 0.0294^n^ | 0.0303^n^ | 0.0524^n^ | -0.154 |
| P36 | LM-KD | 0.2492^a^ | 0.1489^a^ | 0.1313^a^ | 0.1024^a^ | 0.2496^a^ | 0.2024^a^ | 0.1884^a^ | 0.1769^a^ | 0.1967^a^ | 0.184^a^ | 0.1714^a^ | 0.1296^a^ | 0.2571^a^ | 0.0325^n^ | 0.0033^n^ | -0.0348^n^ | 0.149 |
| P37 | LM-EW | 0.2232^a^ | 0.1474^a^ | 0.131^a^ | 0.1034^a^ | 0.2483^a^ | 0.2153^a^ | 0.1998^a^ | 0.1893^a^ | 0.2135^a^ | 0.1998^a^ | 0.1882^a^ | 0.1476^a^ | 0.1687^a^ | -0.0041^n^ | -0.029^n^ | -0.0662^c^ | 0.149 |
| P38 | N.Oct | -0.1243^a^ | -0.1596^a^ | -0.1561^a^ | -0.1294^a^ | -0.2221^a^ | -0.2403^a^ | -0.2302^a^ | -0.2125^a^ | -0.1951^a^ | -0.1979^a^ | -0.199^a^ | -0.1698^a^ | 0.0601^c^ | 0.0015^n^ | 0.0015^n^ | 0.0361^n^ | -0.138 |
| P39 | GRAVY | 0.1372^a^ | 0.1703^a^ | 0.1685^a^ | 0.1425^a^ | 0.2087^a^ | 0.2273^a^ | 0.2325^a^ | 0.2014^a^ | 0.1319^a^ | 0.1194^a^ | 0.1071^b^ | 0.0816^c^ | 0.0311^n^ | 0.1068^a^ | 0.103^b^ | 0.0837^b^ | 0.139 |
| P40 | NH-HW | -0.1144^a^ | -0.1651^a^ | -0.1624^a^ | -0.139^a^ | -0.1936^a^ | -0.2225^a^ | -0.2251^a^ | -0.1981^a^ | -0.1541^a^ | -0.1487^a^ | -0.1357^a^ | -0.1127^b^ | 0.0199^n^ | -0.0808^b^ | -0.0784^c^ | -0.0592^n^ | -0.133 |
| P41 | N.Oct-IF | -0.1793^a^ | -0.1394^a^ | -0.1358^a^ | -0.111^a^ | -0.2421^a^ | -0.224^a^ | -0.2139^a^ | -0.1954^a^ | -0.1294^a^ | -0.1303^a^ | -0.1291^a^ | -0.1064^b^ | -0.1025^a^ | -0.0094^n^ | -0.0095^n^ | 0.0217^n^ | -0.127 |
| P42 | N.IF | -0.0314^n^ | -0.1519^a^ | -0.1502^a^ | -0.1249^a^ | -0.1592^a^ | -0.2178^a^ | -0.2101^a^ | -0.1958^a^ | -0.2065^a^ | -0.2103^a^ | -0.2119^a^ | -0.1752^a^ | 0.2243^a^ | 0.0011^n^ | -0.0007^n^ | 0.0297^n^ | -0.126 |
| P43 | NH-UH | -0.1099^a^ | -0.1595^a^ | -0.1562^a^ | -0.1338^a^ | -0.2089^a^ | -0.2359^a^ | -0.2344^a^ | -0.2074^a^ | -0.1542^a^ | -0.1496^a^ | -0.1351^a^ | -0.1159^b^ | 0.0667^c^ | -0.0385^n^ | -0.0394^n^ | -0.0224^n^ | -0.125 |
| P44 | H-MF | -0.1121^a^ | -0.1418^a^ | -0.1388^a^ | -0.1159^a^ | -0.2127^a^ | -0.232^a^ | -0.225^a^ | -0.1952^a^ | -0.1371^a^ | -0.1374^a^ | -0.1311^a^ | -0.1175^b^ | 0.0539^n^ | -0.004^n^ | -0.0062^n^ | 0.0116^n^ | -0.119 |
| P45 | a3vSA | 0.0059^n^ | 0.0968^a^ | 0.0959^a^ | 0.069^a^ | 0.1208^a^ | 0.1639^a^ | 0.1643^a^ | 0.1386^a^ | 0.0972^b^ | 0.0855^c^ | 0.0757^c^ | 0.0407^n^ | -0.192^a^ | -0.0091^n^ | -0.0065^n^ | -0.0281^n^ | 0.069 |
| P46 | A | 0.1206^a^ | 0.0751^a^ | 0.0739^a^ | 0.0636^a^ | 0.1296^a^ | 0.1028^a^ | 0.1076^a^ | 0.0936^b^ | 0.0519^n^ | 0.039^n^ | 0.0294^n^ | 0.0212^n^ | 0.1272^a^ | 0.0495^n^ | 0.0479^n^ | 0.0401^n^ | 0.0559 |
| P47 | A II | -0.3099^a^ | -0.2156^a^ | -0.207^a^ | -0.1907^a^ | -0.2688^a^ | -0.2257^a^ | -0.2192^a^ | -0.2156^a^ | -0.2897^a^ | -0.2785^a^ | -0.2825^a^ | -0.2543^a^ | -0.3563^a^ | -0.1471^a^ | -0.1245^a^ | -0.1032^b^ | -0.231 |
| P48 | Instability | 0.009^n^ | -0.067^a^ | -0.066^a^ | -0.0663^a^ | -0.0482^n^ | -0.0801^b^ | -0.0873^b^ | -0.0735^c^ | 0.0456^n^ | 0.046^n^ | 0.0582^n^ | 0.0518^n^ | 0.0315^n^ | -0.1387^a^ | -0.138^a^ | -0.1488^a^ | -0.049 |
| P49 | Instability II | -0.099^a^ | -0.1205^a^ | -0.1163^a^ | -0.1093^a^ | -0.144^a^ | -0.1499^a^ | -0.1532^a^ | -0.1369^a^ | -0.0095^n^ | -0.0064^n^ | 0.0032^n^ | 0.0064^n^ | -0.1106^a^ | -0.1702^a^ | -0.1618^a^ | -0.1626^a^ | -0.102 |
| P50 | Depth | -0.0006^n^ | -0.078^a^ | -0.0754^a^ | -0.0547^b^ | -0.1229^a^ | -0.16^a^ | -0.1591^a^ | -0.1409^a^ | -0.0491^n^ | -0.0428^n^ | -0.0294^n^ | 0.0024^n^ | 0.1998^a^ | 0.0325^n^ | 0.0307^n^ | 0.0439^n^ | -0.049 |
| P51 | Angle | -0.0655^a^ | -0.0732^a^ | -0.0744^a^ | -0.0614^a^ | -0.1096^a^ | -0.1131^a^ | -0.1126^a^ | -0.1025^a^ | -0.0342^n^ | -0.0381^n^ | -0.0535^n^ | -0.0375^n^ | -0.0122^n^ | -0.0232^n^ | -0.0147^n^ | -0.0019^n^ | -0.045 |
| P52 | α-B | -0.1621^a^ | -0.1046^a^ | -0.102^a^ | -0.0888^a^ | -0.138^a^ | -0.1118^a^ | -0.1127^a^ | -0.1072^a^ | -0.1307^a^ | -0.1204^a^ | -0.128^a^ | -0.1052^b^ | -0.2204^c^ | -0.0926^b^ | -0.0772^c^ | -0.0625^c^ | -0.117 |
| P53 | N.α-B | 0.102^a^ | 0.0322^n^ | 0.0214^n^ | 0.0246^n^ | 0.0775^b^ | 0.043^n^ | 0.0314^n^ | 0.029^n^ | 0.0243^n^ | 0.0283^n^ | 0.0098^n^ | 0.0269^n^ | 0.1434^c^ | 0.0027^n^ | -0.0016^n^ | -0.0031^n^ | 0.02 |
| P54 | Cα-B | -0.0546^b^ | -0.015^n^ | -0.0233^n^ | -0.0249^n^ | -0.0601^c^ | -0.049^n^ | -0.0593^c^ | -0.0525^n^ | 0.0085^n^ | 0.0101^n^ | -0.0077^n^ | -0.0104^n^ | -0.0929^b^ | 0.0054^n^ | 0.0065^n^ | -0.0042^n^ | -0.017 |
| P55 | CC2 | -0.3813^a^ | -0.3184^a^ | -0.3128^a^ | -0.2875^a^ | -0.3647^a^ | -0.3371^a^ | -0.3311^a^ | -0.3031^a^ | -0.3726^a^ | -0.3626^a^ | -0.3546^a^ | -0.3343^a^ | -0.3834^a^ | -0.246^a^ | -0.2402^a^ | -0.2172^a^ | -0.322 |
| P56 | CC3 | -0.1776^a^ | -0.1537^a^ | -0.1526^a^ | -0.1407^a^ | -0.1695^a^ | -0.1567^a^ | -0.155^a^ | -0.1319^a^ | -0.0839^a^ | -0.0784^a^ | -0.069^n^ | -0.0654^n^ | -0.2429^a^ | -0.2056^a^ | -0.2122^a^ | -0.2082^a^ | -0.142 |
| P57 | AGG-T | -0.066^a^ | -0.0027^n^ | -0.0107^n^ | -0.0262^n^ | -0.0053^n^ | 0.0257^n^ | 0.0208^n^ | -0.0094^n^ | 0.0152^n^ | 0.0098^n^ | -0.0164^n^ | -0.0243^n^ | -0.1974^a^ | -0.068^c^ | -0.0686^c^ | -0.0706^c^ | -0.029 |
| P58 | DSB | 0.0435^a^ | 0.0662^a^ | 0.0632^a^ | 0.0588^a^ | 0.0924^a^ | 0.1066^a^ | 0.1059^a^ | 0.1036^a^ | 0.0392^n^ | 0.0415^n^ | 0.0287^n^ | 0.0173^n^ | -0.0247^n^ | 0.0125^n^ | 0.0093^n^ | 0.0093^n^ | 0.04 |

"a" represents extremely significance (*p* < 0.001); "b" represents very significance (*p* < 0.01); "c" represents significance (*p* < 0.05); "n" represents no significance; "AVCC" was the average valid correlation coefficient of each parameter. The color of yellow represents a positive correlation with MIC; blue represents a negative correlation with MIC; gray-black represents no correlation; brown represents invalid correlation.

**Supplementary Table S4** The minimum (Min) and maximum (Max) values of P1-P45.

| Parameter | Min | Max | Parameter | Min | Max |
| --- | --- | --- | --- | --- | --- |
| P1 | -3 | 23 | P24 | -1.25 | 0.8 |
| P2 | 3.22 | 13.18 | P25 | 0 | 0.57 |
| P3 | 6 | 50 | P26 | 0.12 | 0.57 |
| P4 | 0.22 | 8.33 | P27 | -26.01 | 15.24 |
| P5 | 6.2 | 53.66 | P28 | -6.47 | 23.07 |
| P6 | 1.92 | 73.53 | P29 | 0.1 | 0.57 |
| P7 | 0 | 8.33 | P30 | -1.99 | 1.71 |
| P8 | 0.32 | 8.33 | P31 | 0.11 | 0.54 |
| P9 | 0.22 | 8.33 | P32 | -1.05 | 0.64 |
| P10 | 0 | 1.19 | P33 | -76.1 | 38.4 |
| P11 | 0.27 | 8.33 | P34 | -2.48 | 6.44 |
| P12 | 0 | 8.33 | P35 | 1.48 | 29.9 |
| P13 | 0.26 | 26.2 | P36 | 0 | 0.57 |
| P14 | -4 | 47.7 | P37 | 0.11 | 0.57 |
| P15 | -203 | 40.52 | P38 | -0.76 | 1.81 |
| P16 | -6.08 | 70.95 | P39 | -2.62 | 2.05 |
| P17 | 0.18 | 48.96 | P40 | 0.17 | 2.19 |
| P18 | 0 | 9.6 | P41 | -31 | 128.56 |
| P19 | -2.64 | 19.35 | P42 | -80.88 | 75.57 |
| P20 | 0.14 | 28.86 | P43 | -0.17 | 0.63 |
| P21 | 0.6 | 71.78 | P44 | -47.04 | 69.23 |
| P22 | -0.14 | 0.71 | P45 | -0.67 | 0.86 |
| P23 | 0.52 | 33.48 | - | - | - |

**Supplementary Table S5** Correlation between MIC and MD index, SVM, RF, DA algorithms.

| Dataset | R(MD) | R(SVM) | R(RF) | R(DA) |
| --- | --- | --- | --- | --- |
| A1 | -0.4515^a^ | -0.2124^a^ | -0.3065^a^ | -0.2985^a^ |
| A2 | -0.3571^a^ | -0.0812^a^ | -0.1861^a^ | -0.1733^a^ |
| A3 | -0.3433^a^ | -0.0809^a^ | -0.1751^a^ | -0.1682^a^ |
| A4 | -0.3084^a^ | -0.0815^a^ | -0.154^a^ | -0.1578^a^ |
| E1 | -0.4996^a^ | -0.1676^a^ | -0.2525^a^ | -0.2438^a^ |
| E2 | -0.4605^a^ | -0.1045^a^ | -0.1935^a^ | -0.1831^a^ |
| E3 | -0.4484^a^ | -0.1035^a^ | -0.1869^a^ | -0.177^a^ |
| E4 | -0.424^a^ | -0.1016^a^ | -0.1697^a^ | -0.1739^a^ |
| P1 | -0.4175^a^ | -0.0662^n^ | -0.26^a^ | -0.2912^a^ |
| P2 | -0.405^a^ | -0.0496^n^ | -0.2432^a^ | -0.2778^a^ |
| P3 | -0.3998^a^ | -0.0473^n^ | -0.2334^a^ | -0.274^a^ |
| P4 | -0.3667^a^ | -0.0615^n^ | -0.2052^a^ | -0.259^a^ |
| S1 | -0.3969^a^ | -0.3408^a^ | -0.3878^a^ | -0.3581^a^ |
| S2 | -0.1487^a^ | -0.0732^b^ | -0.1335^a^ | -0.0757^b^ |
| S3 | -0.1228^a^ | -0.0741^b^ | -0.1138^a^ | -0.0679^b^ |
| S4 | -0.0758^b^ | -0.0706^b^ | -0.0961^a^ | -0.0558^n^ |
| AVCC | -0.352 | -0.093 | -0.206 | -0.202 |

R(MD), R(SVM), R(RF) and R(DA) represent the Spearman correlation coefficients between the MD Index, SVM, RF, DA and MIC values, respectively. a, p < 0.001; b, p < 0.01; c, p < 0.05; n, p ≥ 0.05.

**Supplementary Table S6** The cutoff values of 59 parameters.

| Parameter | Name | Cutoff value | Parameter | Name | Cutoff value |
| --- | --- | --- | --- | --- | --- |
| P1 | Net Charge | ≥ 5 | P31 | MF-LM | ≤ 0.26 |
| P2 | pI | ≥ 11 | P32 | H | ≤ 0 |
| P3 | Length | 15-25 | P33 | G2 | ≤ -5 |
| P4 | MF-NLM | ≤ 1.3 | P34 | Boman | ≥ 1.5 |
| P5 | α-helix | ≥ 19.5 | P35 | TM | ≥ 8 |
| P6 | HW-H | ≥ 20 | P36 | KD-LM | ≤ 0.24 |
| P7 | HW-NLM | ≤ 1.3 | P37 | EW-LM | ≤ 0.26 |
| P8 | WW-NLM | ≤ 1.3 | P38 | N.Oct | ≥ 0.5 |
| P9 | UH-NLM | ≤ 1.3 | P39 | G | ≤ -0.2 |
| P10 | HM | ≥ 0.7 | P40 | HW-NH | ≥ 1 |
| P11 | EW-NLM | ≤ 1.3 | P41 | N.Oct-IF | ≥ 40 |
| P12 | KD-NLM | ≤ 1.3 | P42 | N.IF | ≥ 10 |
| P13 | EW-HM | ≥ 9 | P43 | UH-NH | ≥ 0.1 |
| P14 | Oct-IF | ≥ 9.5 | P44 | MF-H | ≥ -1.5 |
| P15 | BM2 | ≤ -30 | P45 | a3vSA | ≤ 0.2 |
| P16 | Oct | ≥ 13.5 | P46 | Aliphatic | 80-120 |
| P17 | MF-HM | ≥ 25 | P47 | Aliphatic II | 18-30 |
| P18 | UH-HM | ≥ 4.5 | P48 | Instability | 0-40 |
| P19 | UH-H | ≥ 3.5 | P49 | Instability II | 0-80 |
| P20 | HW-HM | ≥ 11.5 | P50 | Depth | 14-16 |
| P21 | KD-HM | ≥ 29.5 | P51 | Angle | 85-95 |
| P22 | N.Charge | ≥ 0.2 | P52 | α-B | ≤ 5 |
| P23 | WW-HM | ≥ 13.5 | P53 | Nα-B | ≤ 20.5 |
| P24 | Disordering | ≤ 0 | P54 | Cα-B | = 0 |
| P25 | HW-LM | ≤ 0.24 | P55 | CC2 | ≤ 3 |
| P26 | WW-LM | ≤ 0.26 | P56 | CC3 | = 0 |
| P27 | H2 | ≤ 0 | P57 | AGG-T | ≤ 0.5 |
| P28 | IF(sum) | ≥ 2.5 | P58 | DSB | ≤ 0.5 |
| P29 | UH-LM | ≤ 0.26 | P59 | α-Helix II | ≥ 0 |
| P30 | MF-NH | ≥ -0.1 | - | - | - |

**Supplementary Table S7** The potential AMPs screened by MultiDS screening system.

| No. | MD | C | pI | L | Cs | EV | As | Ds | S2 | SVM | RF | DA* |
| --- | --- | --- | --- | --- | --- | --- | --- | --- | --- | --- | --- | --- |
| S1 | 77.43 | 8 | 12 | 25 | 21 | 0.47 | 41.9 | 0 | α | 0.97 | 0.96 | 1 |
| S2 | 77.3 | 8 | 11 | 24 | 21 | 0.43 | 41.9 | 0 | α | 0.91 | 0.89 | 0.96 |
| S3 | 77.22 | 10 | 12 | 25 | 21 | 0.45 | 46.4 | 0 | α | 0.92 | 0.94 | 0.99 |
| S4 | 76.64 | 7 | 12 | 25 | 21 | 0.42 | 38.5 | 0 | α | 0.96 | 0.95 | 1 |
| S5 | 76.63 | 7 | 12 | 23 | 22 | 0.15 | 41.7 | 0 | α | 0.91 | 0.98 | 1 |
| S6 | 76.6 | 9 | 12 | 25 | 19 | 2.4 | 42.9 | 0 | α | 0.99 | 0.98 | 1 |
| S7 | 76.46 | 6 | 12 | 24 | 22 | 1 | 38.5 | 0 | α | 0.85 | 0.88 | 0.94 |
| S8 | 76.39 | 9 | 12 | 24 | 19 | 2.2 | 44.4 | 0 | α | 0.97 | 0.94 | 1 |
| S9 | 76.39 | 7 | 11 | 25 | 23 | 0.13 | 45.2 | 0 | α | 0.92 | 0.98 | 0.96 |
| S10 | 76.22 | 8 | 12 | 24 | 23 | 0.43 | 44 | 0 | α | 0.93 | 0.88 | 0.96 |
| S11 | 76.16 | 9 | 11 | 23 | 20 | 0.71 | 41.7 | 0 | α | 0.93 | 0.96 | 0.99 |
| S12 | 76.15 | 9 | 12 | 25 | 21 | 0.45 | 46.9 | 0 | α | 0.88 | 0.92 | 0.94 |
| S13 | 76.06 | 7 | 12 | 23 | 20 | 0.71 | 41.7 | 0 | α | 0.99 | 0.97 | 0.99 |
| S14 | 75.96 | 7 | 11 | 23 | 22 | 0.67 | 40 | 0 | α | 0.89 | 0.93 | 0.95 |
| S15 | 75.85 | 7 | 12 | 23 | 20 | 1.3 | 46.2 | 0 | α | 0.95 | 0.96 | 1 |
| S16 | 75.72 | 8 | 12 | 24 | 21 | 0.45 | 42.9 | 0 | α | 0.99 | 0.98 | 1 |
| S17 | 75.61 | 8 | 12 | 23 | 21 | 0.44 | 42.9 | 0 | α | 0.99 | 0.97 | 1 |
| S18 | 75.54 | 8 | 12 | 21 | 19 | 2.7 | 41.7 | 0 | α | 0.94 | 0.86 | 0.98 |
| S19 | 75.33 | 7 | 12 | 23 | 22 | 0.28 | 43.5 | 0 | α | 0.98 | 0.93 | 1 |
| S20 | 75.25 | 6 | 11 | 24 | 22 | 0.25 | 40 | 0 | α | 0.99 | 0.99 | 0.99 |
| S21 | 75.22 | 6 | 12 | 25 | 20 | 1.5 | 42.4 | 0 | α | 0.94 | 0.88 | 1 |
| S22 | 75.18 | 6 | 11 | 25 | 20 | 1.5 | 37.1 | 0 | α | 0.96 | 0.9 | 1 |
| S23 | 74.99 | 7 | 11 | 24 | 23 | 0.08 | 39.3 | 0 | α | 0.98 | 0.96 | 1 |
| S24 | 74.74 | 8 | 13 | 25 | 21 | 0.49 | 46.4 | 0 | α | 0.96 | 0.98 | 1 |
| S25 | 74.72 | 6 | 11 | 25 | 22 | 0.26 | 44.4 | 0 | α | 0.99 | 1 | 1 |
| S26 | 74.71 | 10 | 13 | 25 | 24 | 0.05 | 40.6 | 0 | α | 0.96 | 1 | 1 |
| S27 | 74.7 | 9 | 12 | 25 | 24 | 0.05 | 42.3 | 0 | α | 1 | 0.98 | 1 |
| S28 | 74.62 | 6 | 12 | 25 | 20 | 1.1 | 37 | 0 | α | 0.94 | 0.91 | 1 |
| S29 | 74.61 | 8 | 12 | 24 | 20 | 1.7 | 42.3 | 0 | α | 0.9 | 0.93 | 0.91 |
| S30 | 74.44 | 9 | 12 | 25 | 18 | 5.3 | 40.7 | 0 | α | 0.86 | 0.89 | 1 |
| S31 | 74.4 | 9 | 13 | 25 | 19 | 7.6 | 42.9 | 0 | α | 0.91 | 0.97 | 0.99 |
| S32 | 74.35 | 9 | 12 | 25 | 23 | 0.1 | 40.7 | 0 | α | 0.98 | 0.99 | 0.96 |
| S33 | 74.34 | 8 | 12 | 25 | 22 | 0.3 | 41.4 | 0 | α | 0.93 | 0.94 | 0.97 |
| S34 | 74.34 | 8 | 13 | 25 | 21 | 0.49 | 42.9 | 0 | α | 0.96 | 0.99 | 0.97 |
| S35 | 74.27 | 7 | 12 | 23 | 23 | 0.42 | 42.3 | 0 | α | 0.94 | 0.9 | 0.92 |
| S36 | 74.26 | 8 | 12 | 25 | 24 | 0.06 | 41.9 | 0 | α | 0.97 | 0.88 | 0.99 |
| S37 | 74.26 | 8 | 12 | 25 | N | — | 42.9 | 0 | α | 0.93 | 0.91 | 0.94 |
| S38 | 74.25 | 7 | 12 | 24 | 20 | 2.4 | 42.3 | 0 | α | 0.87 | 0.96 | 0.99 |
| S39 | 74.17 | 7 | 11 | 25 | 19 | 2.1 | 41.4 | 0 | α | 0.91 | 1 | 0.87 |
| S40 | 74.14 | 8 | 12 | 22 | 21 | 0.41 | 45.5 | 0 | α | 0.95 | 0.87 | 0.99 |
| S41 | 74.08 | 8 | 13 | 25 | 20 | 1.5 | 44.8 | 0 | α | 0.94 | 0.92 | 0.99 |
| S42 | 74.06 | 5 | 12 | 25 | N | — | 40 | 0 | α | 0.87 | 0.86 | 0.96 |
| S43 | 74.03 | 7 | 12 | 20 | 20 | 0.86 | 40.9 | 0 | α | 0.92 | 0.93 | 0.99 |
| S44 | 74.01 | 8 | 12 | 22 | 21 | 0.41 | 45.5 | 0 | α | 0.92 | 0.85 | 0.99 |
| S45 | 74 | 8 | 12 | 24 | 22 | 0.44 | 39.3 | 0 | α | 0.88 | 0.97 | 0.98 |
| S46 | 73.91 | 9 | 13 | 25 | 21 | 0.99 | 43.3 | 0 | α | 0.93 | 0.98 | 1 |
| S47 | 73.88 | 7 | 11 | 25 | 23 | 0.44 | 46.4 | 0 | α | 0.95 | 0.89 | 0.92 |
| S48 | 73.86 | 7 | 12 | 25 | 20 | 1.2 | 37.9 | 0 | α | 0.96 | 0.96 | 1 |
| S49 | 73.84 | 8 | 13 | 22 | 23 | 0.11 | 43.5 | 0 | α | 0.98 | 0.93 | 1 |
| S50 | 73.8 | 8 | 12 | 24 | 19 | 2.2 | 42.3 | 0 | α | 0.89 | 0.96 | 0.98 |
| S51 | 73.79 | 8 | 11 | 25 | 20 | 1.1 | 40.7 | 0 | α | 0.86 | 0.99 | 0.99 |
| S52 | 73.78 | 9 | 12 | 24 | 22 | 0.21 | 46.2 | 0 | α | 0.94 | 0.99 | 1 |
| S53 | 73.75 | 8 | 13 | 24 | 20 | 1.5 | 45.8 | 0 | α | 0.94 | 0.97 | 0.99 |
| S54 | 73.65 | 7 | 11 | 25 | 20 | 2.5 | 44 | 0 | α | 0.92 | 0.99 | 1 |
| S55 | 73.64 | 5 | 12 | 25 | 20 | 3.9 | 38.7 | 0 | α | 0.95 | 0.88 | 0.98 |
| S56 | 73.63 | 7 | 12 | 25 | 21 | 1.3 | 41.4 | 0 | α | 0.89 | 0.95 | 0.99 |
| S57 | 73.61 | 9 | 13 | 24 | 21 | 0.95 | 44.8 | 0 | α | 0.96 | 0.99 | 1 |
| S58 | 73.46 | 7 | 13 | 24 | 21 | 1.3 | 39.3 | 0 | α | 0.98 | 0.95 | 1 |
| S59 | 73.36 | 7 | 11 | 25 | 22 | 0.3 | 42.9 | 0 | α | 0.95 | 0.9 | 0.93 |
| S60 | 73.35 | 8 | 12 | 25 | 23 | 0.13 | 42.3 | 0 | α | 0.98 | 0.91 | 1 |

* MD: MD index, C: net charge, pI: isoelectric point, L: length, Cs: similarity in the CAMPR3 database, EV: E.Value, As: similarity in the APD3 database, Ds: similarity in the DBAASP database, S2: Secondary structure of peptides predicted by APD3 database, α: Alpha helical structure, SVM: support vector machine, RF: random forest, DA: discriminant analysis.

## REFERENCES

Boman, H.G. (2003). Antibacterial peptides: basic facts and emerging concepts. *J. Intern. Med.* 254**,** 197-215. doi: 10.1046/j.1365-2796.2003.01228.x.

Chou, P.Y., and Fasman, G.D. (1974a). Conformational parameters for amino acids in helical, beta-sheet, and random coil regions calculated from proteins. *Biochemistry* 13**,** 211-222. doi: 10.1021/bi00699a001.

Chou, P.Y., and Fasman, G.D. (1974b). Prediction of protein conformation. *Biochemistry* 13**,** 222-245. doi: 10.1021/bi00699a002.

Conchillo-Solé, O., de Groot, N.S., Avilés, F.X., Vendrell, J., Daura, X., and Ventura, S. (2007). AGGRESCAN: a server for the prediction and evaluation of "hot spots" of aggregation in polypeptides. *BMC Bioinformatics* 8(1)**,** 65. doi: 10.1186/1471-2105-8-65.

de Groot, N.S., Castillo, V., Graña-Montes, R., and Ventura, S. (2012). AGGRESCAN: method, application, and perspectives for drug design. *Methods Mol. Biol.* 819**,** 199-220. doi: 10.1007/978-1-61779-465-0_14.

Eisenberg, D., Schwarz, E., Komaromy, M., and Wall, R. (1984a). Analysis of membrane and surface protein sequences with the hydrophobic moment plot. *J. Mol. Biol.* 179**,** 125-142. doi: 10.1016/0022-2836(84)90309-7.

Eisenberg, D., Weiss, R.M., and Terwilliger, T.C. (1982). The helical hydrophobic moment: a measure of the amphiphilicity of a helix. *Nature* 229**,** 371-374. doi: 10.1038/299371a0.

Eisenberg, D., Weiss, R.M., and Terwilliger, T.C. (1984b). The hydrophobic moment detects periodicity in protein hydrophobicity. *Proc. Natl. Acad. Sci. U S A.* 81**,** 140-144. doi: 10.1073/pnas.81.1.140.

Guruprasad, K., Reddy, B.V., and Pandit, M.W. (1990). Correlation between stability of a protein and its dipeptide composition: a novel approach for predicting in vivo stability of a protein from its primary sequence. *Protein Eng.* 4**,** 155-161. doi: 10.1093/protein/4.2.155.

Hessa, T., Kim, H., Bihlmaier, K., Lundin, C., Boekel, J., Andersson, H., et al. (2005). Recognition of transmembrane helices by the endoplasmic reticulum translocon. *Nature* 433**,** 377-381. doi: 10.1038/nature03216.

Ikai, A. (1980). Thermostability and aliphatic index of globular proteins. *J. Biochem.* 88**,** 1895-1898. doi: 10.1093/oxfordjournals.jbchem.a133168.

Koehler, J., Woetzel, N., Staritzbichler, R., Sanders, C.R., and Meiler, J. (2009). A unified hydrophobicity scale for multispan membrane proteins. *Proteins* 76(1)**,** 13-29. doi: 10.1002/prot.22315.

Kozlowski, L.P. (2016). IPC - Isoelectric Point Calculator. *Biol. Direct.* 11(1)**,** 55. doi: 10.1186/s13062-016-0159-9.

Kyte, J., and Doolittle, R.F. (1982). A simple method for displaying the hydropathic character of a protein. *J. Mol. Biol.* 157**,** 105-132. doi: 10.1016/0022-2836(82)90515-0.

Moon, C.P., and Fleming, K.G. (2011). Side-chain hydrophobicity scale derived from transmembrane protein folding into lipid bilayers. *Proc. Natl. Acad. Sci. U S A.* 108(25)**,** 10174-10177. doi: 10.1073/pnas.1103979108.

Moore, D.S. (1985). Amino acid and peptide net charges: A simple calculational procedure. *Biochem. Mol. Biol. Edu.* 13(1)**,** 10-11. doi: 10.1016/0307-4412(85)90114-1.

Pirtskhalava, M., Gabrielian, A., Cruz, P., Griggs, H.L., Squires, R.B., Hurt, D.E., et al. (2016). DBAASP v.2: an enhanced database of structure and antimicrobial/cytotoxic activity of natural and synthetic peptides. *Nucleic. Acids. Res.* 44(D1)**,** D1104-D1112. doi: 10.1093/nar/gkv1174.

Radzicka, A., and Wolfenden, R. (1988). Comparing the polarities of the amino acids: side-chain distribution coefficients between the vapor phase, cyclohexane, 1-octanol, and neutral aqueous solution. *Biochemistry* 27**,** 1664-1670. doi: doi.org/10.1021/bi00405a042.

Senes, A., Chadi, D.C., Law, P.B., Walters, R.F., Nanda, V., and Degrado, W.F. (2007). E(z), a depth-dependent potential for assessing the energies of insertion of amino acid side-chains into membranes: derivation and applications to determining the orientation of transmembrane and interfacial helices. *J. Mol. Biol.* 366(2)**,** 436-448. doi: 10.1016/j.jmb.2006.09.020.

Snider, C., Jayasinghe, S., Hristova, K., and White, S.H. (2009). MPEx: a tool for exploring membrane proteins. *Protein. Sci.* 18(12)**,** 2624-2628. doi: 10.1002/pro.256.

Uversky, V.N., Gillespie, J.R., and Fink, A.L. (2000). Why are "natively unfolded" proteins unstructured under physiologic conditions? *Proteins* 41**,** 415-427. doi: 10.1002/1097-0134(20001115)41:3<415::aid-prot130>3.0.co;2-7.

Vishnepolsky, B., and Pirtskhalava, M. (2014). Prediction of linear cationic antimicrobial peptides based on characteristics responsible for their interaction with the membranes. *J. Chem. Inf. Model.* 54(5)**,** 1512-1523. doi: 10.1021/ci4007003.

Wimley, W.C., and White, S.H. (1996). Experimentally determined hydrophobicity scale for proteins at membrane interfaces. *Nat. Struct. Biol.* 3**,** 842-848. doi: 10.1038/nsb1096-842.
